# Single-cell analysis of the postnatal dorsal V-SVZ reveals a role for Bmpr1a signaling in silencing pallial germinal activity

**DOI:** 10.1126/sciadv.abq7553

**Published:** 2023-05-05

**Authors:** Guillaume Marcy, Louis Foucault, Elodie Babina, Timothy Capeliez, Emeric Texeraud, Stefan Zweifel, Christophe Heinrich, Hector Hernandez-Vargas, Carlos Parras, Denis Jabaudon, Olivier Raineteau

**Affiliations:** ^1^ Univ Lyon, Université Claude Bernard Lyon 1, Inserm, Stem Cell and Brain Research Institute U1208, 69500 Bron, France.; ^2^ Univ Lyon, Université Claude Bernard Lyon 1, Bioinformatic Platform of the Labex Cortex, 69008 Lyon, France.; ^3^ Cancer Research Centre of Lyon (CRCL), INSERM U 1052, CNRS UMR 5286, UCBL1, Université de Lyon, Centre Léon Bérard, 28 rue Laennec, 69373 Lyon Cedex 08, France.; ^4^ Paris Brain Institute, Sorbonne Université, Inserm U1127, CNRS UMR 7225, Hôpital Pitié-Salpêtrière, 75013 Paris, France.; ^5^ Department of Basic Neurosciences, University of Geneva, Geneva, Switzerland.; ^6^ Clinic of Neurology, Geneva University Hospital, Geneva, Switzerland.

## Abstract

The ventricular-subventricular zone (V-SVZ) is the largest neurogenic region of the postnatal forebrain, containing neural stem cells (NSCs) that emerge from both the embryonic pallium and subpallium. Despite of this dual origin, glutamatergic neurogenesis declines rapidly after birth, while GABAergic neurogenesis persists throughout life. We performed single-cell RNA sequencing of the postnatal dorsal V-SVZ for unraveling the mechanisms leading to pallial lineage germinal activity silencing. We show that pallial NSCs enter a state of deep quiescence, characterized by high bone morphogenetic protein (BMP) signaling, reduced transcriptional activity and Hopx expression, while in contrast, subpallial NSCs remain primed for activation. Induction of deep quiescence is paralleled by a rapid blockade of glutamatergic neuron production and differentiation. Last, manipulation of Bmpr1a demonstrates its key role in mediating these effects. Together, our results highlight a central role of BMP signaling in synchronizing quiescence induction and blockade of neuronal differentiation to rapidly silence pallial germinal activity after birth.

## Teaser

scRNA-Seq reveals lineage-specific events resulting in a rapid closure of glutamatergic neurogenesis within the mouse V-SVZ.

## INTRODUCTION

During development, radial glial (RG) cells residing in the ventricular (VZ) and subventricular zone (SVZ) are multipotent stem cells, generating ependymal cells (ECs), neurons, and glial cells. The production of these cell types is molecularly regulated in space and time. In mammals, excitatory glutamat(GLU)ergic neurons are produced by dorsal (i.e., pallial) RG cells, while inhibitory γ-amino butyric acid (GABA)ergic neurons are generated by more ventral (i.e., subpallial) regions.

While most forebrain neurons are produced before birth, neurogenesis persists in specific brain regions. The ventricular-subventricular zone (V-SVZ) is the largest and most heterogeneous germinal region of the postnatal brain (*1*). The ventral and lateral parts of the V-SVZ derive from the embryonic subpallium, while its dorsal part derives from the pallium (*2*). Ventral and lateral V-SVZ germinal cells are derived from a subset of RG cells, which transiently enter quiescence late embryonically and then gradually reactivate postnatally (*3*, *4*). In particular, these cells [defined as neural stem cells (NSCs) or type B cells] continue producing distinct subtypes of olfactory bulb (OB) interneurons throughout life, most of which are GABAergic.

This exclusive GABAergic neurogenesis contrasts with the pallial origin of at least a subpopulation of V-SVZ NSCs. Within this domain, most RG cells do not undergo a neurogenic-to-gliogenic switch (*5*) but, instead, remain able to generate GLUergic progenitors at early postnatal times (*6*). These progenitors express *Neurog2* and *Eomes*/*Tbr2* and persist until at least 2 months in the mouse (*7*). Although evidences indicate that at least a fraction of these cells contributes to GLUergic neurogenesis within the cortex at birth (*6*), as well as to OB neurogenesis during early postnatal life (*8*) until young adulthood (*9*), they are mostly outnumbered by GABAergic progenitors. Thus, uncharacterized mechanisms ensure the relative abrupt halt in GLUergic neurogenesis shortly after birth, contrasting with the continuity of GABAergic neurogenesis throughout life.

Single-cell RNA sequencing (scRNA-seq) is a powerful approach to unravel the heterogeneity and dynamics of NSCs at adult stages. For instance, prospective isolation of NSCs has been achieved by fluorescence-activated cell sorting based on the expression of selected markers (*10*). Microdissections of the lateral, ventral, and medial V-SVZ followed by Drop-Seq or automated microwell array–based platform have also been achieved (*11*, *12*), providing insights into the transcriptional coding of NSC competence and dynamics. Here, we aimed at complementing these previous studies by focusing on the early postnatal life, a period of transition between embryonic development and adulthood. Furthermore, we focused on a so-far unexplored region of the V-SVZ, i.e., its dorsal compartment, where NSCs of both the pallial and subpallial origin coexist (*1*). This allowed us to unravel the transcriptional hallmarks associated with the rapid closure of GLUergic neurogenesis, while GABAergic neurogenesis persists. We demonstrate that several mechanisms coordinate the rapid silencing of pallial NSCs. These include their transition into deep quiescence and a blockade of neuronal differentiation, which are both regulated by transforming growth factor–β (TGFβ)/bone morphogenetic protein (BMP) signaling through Bmpr1a.

## RESULTS

### Analysis of the postnatal dorsal V-SVZ by large-scale single-cell profiling

**Fig. 1.**
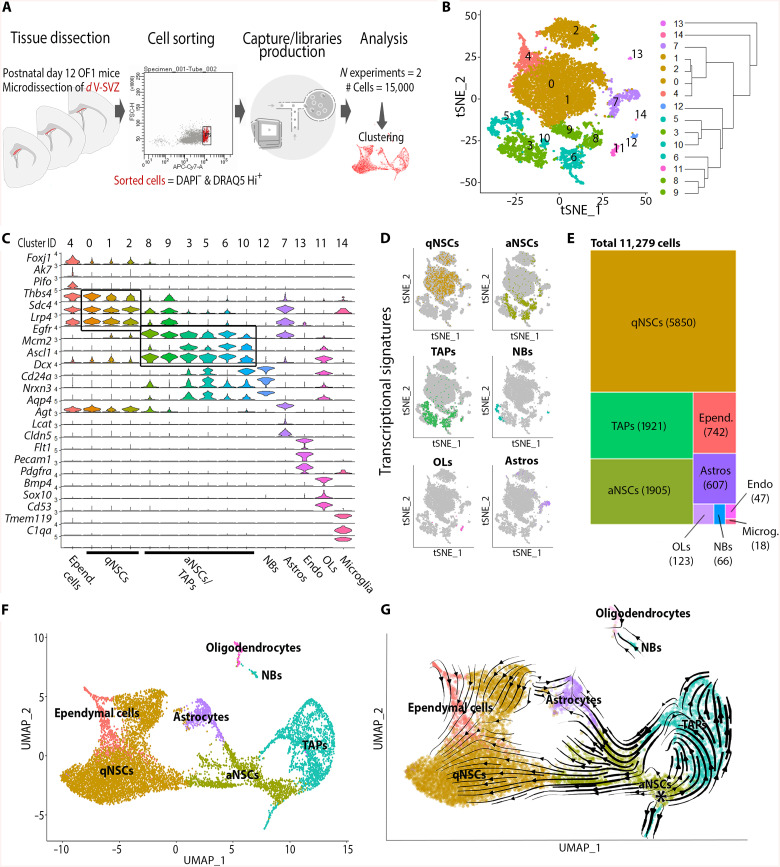
Analysis of the postnatal V-SVZ dorsal domain cellular composition and differentiation dynamics by large-scale single-cell profiling. (**A)** Scheme of the microdissection and experimental workflow. (**B**) t-distributed stochastic neighbor embedding (t-SNE) projections of major cell types. Color coding corresponds to cell types shown in (D) and (E). Note that quiescent NSCs (qNSCs) represent the larger cell clusters and comprise three clusters of different sizes, which have been grouped in the t-SNE plot (brown). (**C**) Violin plots showing known markers for V-SVZ cell types. (**D**) Combined expression of key markers on t-SNEs (one combination of markers per major cell type). (**E**) Treemap representing the proportion of the major cell types present in our dataset. (**F**) Uniform Manifold Approximation and Projection (UMAP) plot depicting the simplified identity annotation. Endothelial cells and microglial cells are not shown. (**G**) RNA velocity analysis highlighting emergence of multiple trajectories from activated NSCs (aNSCs) toward qNSCs, glial cells, and transit-amplifying progenitors (TAPs). The starting point of these trajectories is indicated by an asterix. Epend., ependymal; NBs, neuroblasts; Astros, astrocytes; Endo, endothelial cells; OLs, oligodendroglial lineage cells.

To gain an in-depth overview of the cellular and molecular heterogeneity of the dorsal V-SVZ, we performed large-scale single-cell profiling from the microdissected dorsal wall of the V-SVZ at postnatal day 12 (P12) (Fig. 1A). Following quality control of independent replicates (fig. S1, A and C), we obtained the transcriptome of 11,279 cells (out of >15,000 raw cell counts) by scRNA-seq using 10x Genomics protocol and high coverage (median of about 4500 genes detected per cell).

Successful isolation of the dorsal V-SVZ cells were validated by the absence of cells expressing *Nkx2-1* and *Nkx2-3*, as well as by minimal expression of *Vax1* (67 cells) (*13*). In sharp contrast, expression of more dorsal markers such as *Msx1* (1486 cells), *Gsx2* (1845 cells), and *Emx1* (4082) was detected in a notable proportion of all cells (11.1, 16.3, and 36.1%, respectively). Furthermore, evidence of an absence of contamination by cells of ventral lineages was supported by the consistent detection of four transcripts recently identified to be confined throughout the dorsal lineage (*Rlbp1*, *Gm29260*, *Pax6*, and *Dcc*), while the five gold standard transcripts (*Adgrl3*, *Slit2*, *Ptprn2*, *Rbms1*, and *Sntb1*) delineating the ventral lineage were very lowly expressed (fig. S1D) (*14*).

Downstream clustering revealed 15 distinct clusters (resolution 0.5; Fig. 1B), among which 12 corresponded to cells of the neural lineage. Cell cluster identification was based on the detection of landmark cell type markers, alone (Fig. 1C and fig. S1, E and F) or in combination (Fig. 1D and table S7). Identification of quiescent NSCs (qNSCs) was based on the expression of *Glast* and *Prom1*, as well as the exclusion of *Egfr* and of the EC marker *Foxj1.* Activated NSCs (aNSCs) were assigned by their expression of *Egfr* and *Ascl1*, but exclusion of *Dlx1* and *Dlx2*, whereas transit-amplifying progenitors (TAPs) were defined by an elevated expression of these markers, but low expression of *Sp8*, *Gad1*, and *Gad2*. We observed expression of broad generic markers of immature neurons (*Dcx*, *cd24a*, and *Nrxn3*), both within TAPs (i.e., outlining neuronal commitment; see also fig. S2, A and B) and in a separate cluster corresponding to migrating neuroblasts (NBs). Last, we defined oligodendroglial lineage cells (OLs) on the basis of *Pdgfra* and *Sox10* expression and astrocytes (Astros) on an elevated level of *S100b* and *Aqp4* expression (Fig. 1, C and D). These initial analyses identified qNSCs as the prime cohorts of cells captured within our dataset (52% of all cells), followed by aNSCs and TAPs (17% each). ECs (6.6%) and Astros (5.4%) were lowly represented. The restricted proportion of OLs (1%) and NBs (0.5%) underlined the accuracy of the V-SVZ microdissection, with minimal inclusion of the overlying corpus callosum or of the rostral migratory stream. Last, few non-neural cell types were also present in our dataset (endothelial/mural cells, microglial cells, <0.5%) (Fig. 1E). Together, these findings highlight the precision of our microdissection strategy for careful inspection of dorsal V-SVZ NSCs at distinct stages of activation, as well as of all their neural progenies, i.e., ependymal, neuronal, and glial cells.

### Lineage progression within the postnatal dorsal V-SVZ

We next generated a Uniform Manifold Approximation and Projection (UMAP) plot with simplified identity annotations for qNSCs, aNSCs, and TAPs (all composed of three subclusters) to explore the transcriptional relationship between clusters. qNSCs and aNSCs occupied the center of the UMAP plot, while ECs, neuronal cells, and glial cells appeared as peripheral clusters (Fig. 1F and fig. S2, A and B). To gain insights into the major metabolic processes that define the subpopulation of NSCs and their progeny, the top 20 genes associated with previously described state transitions were overlaid onto the UMAP (*10*). This confirmed the identification of major cell types and revealed parallels with the adult V-SVZ, including (i) the association of dormancy with elevated glycolytic and lipid metabolisms in qNSCs (*15*); (ii) the correlation of ribosomal transcripts with NSC activation; and (iii) the rapid sequence of cell cycle initiation, mitosis, and neuronal differentiation in TAPs (fig. S3, A and B, and table S7). Similar biological processes were highlighted by an overrepresentation analysis performed on genes up- and down-regulated at transitions between these differentiation stages. This analysis highlighted the gradual down-regulation from qNSCs to TAPs of genes involved in pluripotency, gliogenesis, and glycolytic metabolism, as well as the inversely correlated up-regulation of genes involved in ribosome biogenesis and mitosis (fig. S3, C and D).

We next assessed the cell lineage progression within our datasets by performing nonsupervised RNA velocity lineage trajectory reconstruction (*16*). In contrast to adult V-SVZ neurogenesis, in which the generation of olfactory neuronal subtypes initiates from qNSCs (*10*), postnatal aNSCs were the major pool from which multiple trajectories emerged projecting toward (i) qNSCs, (ii) Astros and oligodendroglia, and (iii) TAPs and their neuronal progeny (Fig. 1G). Thus, although important similarities are observed in the NSC differentiation stages and metabolic machineries between the postnatal and adult V-SVZ, important differences exist in lineage progression and entry into quiescence between its dorsal and lateral subregions.

### aNSCs are heterogeneous and primed for lineage differentiation

**Fig. 2.**
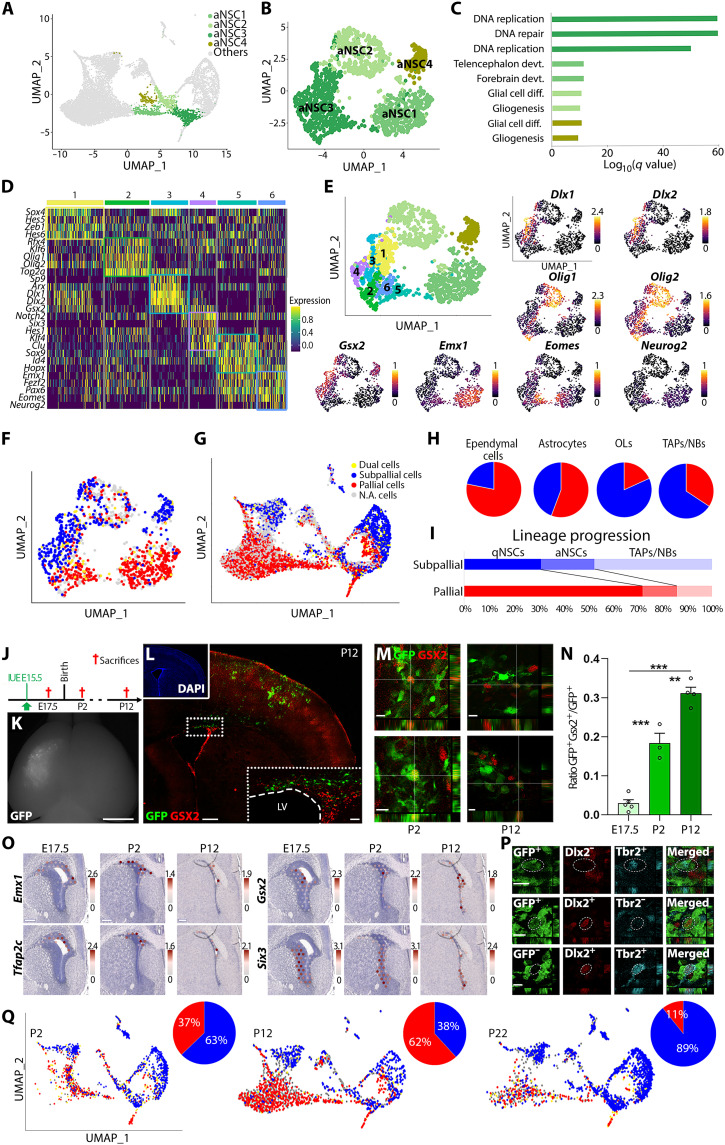
Early priming of pallial and subpallial aNSCs along multiple trajectories. (**A** and **B**) UMAP plots of complete dataset and aNSCs subclusters. (**C**) Bar plot of significantly overrepresented Gene Ontology (GO) terms using genes enriched in aNSCs subclusters. (**D**) Heatmap showing the top five differentially expressed transcription factors (TFs) for each of the six aNSC3 subclusters. (**E**) UMAP plot with identity of aNSC3 subclusters and illustrating feature plots of markers identified in (D). (**F** and **G**) UMAP plots identifying cells expressing pallial (red) or subpallial (blue) transcripts (or both, i.e., dual cells; yellow) within aNSCs (F) or within the entire dataset (G). N.A. cells, not applicable cells. (**H**) Percentage of cells expressing pallial (red) or subpallial (blue) transcripts within NSCs progenies. (**I**) Percentage of qNSCs, aNSCs, and TAPs/NBs in each lineage. (**J** to **N**) Fate map of pallial NSCs by electroporation of a transposon green fluorescent protein (GFP) plasmid at E15.5 (J and K) reveals NSCs of pallial origin acquiring subpallial lineage markers at postnatal stages [here, Gsx2; (L) and (M)], as quantified in (N). DAPI, 4′,6-diamidino-2-phenylindole. (**O**) Spatial transcriptomic (Visium) showing invasion of dorsal V-SVZ territories by subpallial markers (i.e., *Six3* and *Gsx2*) at postnatal times only. (**P**) Postnatal acquisition of subpallial traits by pallial NSCs, and their neuronal progeny is further supported by coexpression of Tbr2 with Dlx2 as well as *GAD67^GFP^* in the dorsal V-SVZ. (**Q**) Analysis of cell proportion at P2, P12, and P22. Scale bars, 2.5 mm (K), 0.5 mm (L), 20 μm (inset, L), 10 μm (M), 250 μm (O), and 10 μm (P). See also tables S1 and S2. **P < 0.01, ***P < 0.001. LV, lateral ventricle.

To further understand aNSC molecular states, we performed a subclustering analysis. This revealed four different clusters of aNSCs, which were segregated in a UMAP plot (Fig. 2, A and B), with aNSC3 spatially corresponding to the “starter cell population” identified in the RNA velocity analysis (see asterisk location in Fig. 1G). To determine the main biological differences between aNSC clusters, we performed Gene Ontology (GO) analysis on genes enriched in each cluster (aNSC1, 522 genes; aNSC2, 337 genes; aNSC3, 504 genes; and aNSC4, 525 genes). Genes related to “proliferation” (GO:0006260, GO:0044770, and GO:0006281) were among those most highly associated with aNSC3, while terms related to “development” (GO:0021537, GO:0030900, and GO:0021987), “differentiation” (GO:0010001, GO:0045685, and GO:0048709), and “gliogenesis” (GO:0042063) were enriched in aNSC1, aNSC2, and aNSC4, respectively (Fig. 2C and table S1).

To gain further insights into this central aNSC3 population (569 cells), we performed additional subclustering at higher resolution, identifying six further clusters. Analysis of the differential expression of key transcription factors (TFs) among these clusters (Fig. 2, D and E, and table S2) indicated that the aNSC3 cluster is composed of a mixture of cells primed for differentiation into different lineages, rather than a homogeneous population of multipotent NSCs. In particular, clusters 1 and 2 expressed TFs associated with oligodendrogenesis (e.g., *Olig1/2*), while clusters 4 and 5 expressed TFs associated with neurogenesis (i.e., *Dlx2* and *Neurog2*, respectively). TFs defining subpallial and pallial lineages segregated across clusters 3/4 and 5/6, respectively, suggesting the coexistence of both lineages within our dorsal V-SVZ datasets (Fig. 2E).

### Cells of the pallial and subpallial lineages coexist within the dorsal V-SVZ and show different dynamics

Expression of markers for either pallial (*Emx1*, *Neurod1*, *Neurod6*, *Neurog2*, and *Tbr1*; i.e., 3862 cells) or subpallial (*Gsx2*, *Dlx6*, *Dlx5*, *Sp9*, *Dlx2*, and *Gad1*; i.e., 2452 cells) lineages confirmed the coexistence of two lineages not only within aNSCs (Fig. 2F) but also more broadly throughout the entire datasets (Fig. 2G). A smaller population of cells expresses both pallial and subpallial markers (“Dual cells”; Fig. 2, F and G), which were not doublets as revealed by their averaged RNA content (fig. S4A), as well as by doublet detection analyses, suggesting lineage transition for a small number of cells. Quantification of the proportion of cells expressing pallial (*Emx1*) and subpallial (*Gsx2*) markers revealed a differential contribution of both lineages to defined cell types, with ECs mainly expressing the pallial marker *Emx1* and Astros having equal proportions, whereas OLs and TAPs/NBs mainly expressing the subpallial marker *Gsx2* (Fig. 2H). Furthermore, comparison of qNSC, aNSC, and TAPs/NB respective proportions between both lineages revealed profound differences. TAPs/NBs were predominant within *Gsx2*^+^ cells (i.e., 47% of the cells, compared to 21% for aNSCs and 31% for qNSCs), while qNSCs were predominant within *Emx1*^+^ cells (i.e., 72% of all cells, with TAPs/NBs representing only 14% of all cells) (Fig. 2I), suggesting different levels of activation/quiescence between cells of the pallial and subpallial lineages. The presence of both lineages within the postnatal dorsal V-SVZ was further confirmed by electroporation of an integrative green fluorescent protein (GFP) plasmid in embryonic day 15.5 (E15.5) pallial NSCs to follow their fate at embryonic and postnatal times (Fig. 2, J to N). Immunostaining for Gsx2 at E17.5 confirmed the rarity (<3%) of GFP^+^ cells expressing this marker within the embryonic pallium. Notably, an increasing percentage of fate-mapped GFP^+^ cells expressed Gsx2 at P2 and P12, confirming the gradual emergence of a population of pallial cells expressing subpallial markers during postnatal life (Fig. 2N), in agreement with a previous report (*17*). This was further supported by Visium spatial transcriptomics (Fig. 2O). While pallial (*Emx1* and *Tfap2c*) and subpallial markers (*Gsx2* and *Six3*) showed strict spatial segregation at E17.5, subpallial markers progressively invaded dorsal V-SVZ at postnatal stages. In addition, colocalization of Dlx2 and/or *GAD67^GFP^* with Tbr2 confirmed that coexpression was not only restricted to NSCs but also included TAPs/NBs (Fig. 2P) in which the proportion of dual cells culminates (i.e., 8% of the cells; fig. S4, B and C).

To confirm these results and explore the dynamic of pallial and subpallial lineages within the dorsal V-SVZ, we produced two additional datasets early after birth (P2) and at weaning (P22) (fig. S1B). Results confirmed the presence of cells of the two lineages at all time points (Fig. 2Q). Pallial cells peaked at P12, because of a pronounced accumulation of cells corresponding to qNSCs (see Fig. 1F), but declined by P22 at the expense of cells of the subpallial lineage. Together, these results highlight the coexistence of cells presenting cardinal features of pallial and subpallial lineages within the postnatal dorsal V-SVZ. Furthermore, our results suggest profound differences in the dynamic of these two lineages, with most pallial cells entering quiescence, while subpallial cells produce a large population of TAPs/NBs.

### Pallial NSCs enter quiescence at postnatal stages within the dorsal V-SVZ and are characterized by *Hopx* expression

**Fig. 3.**
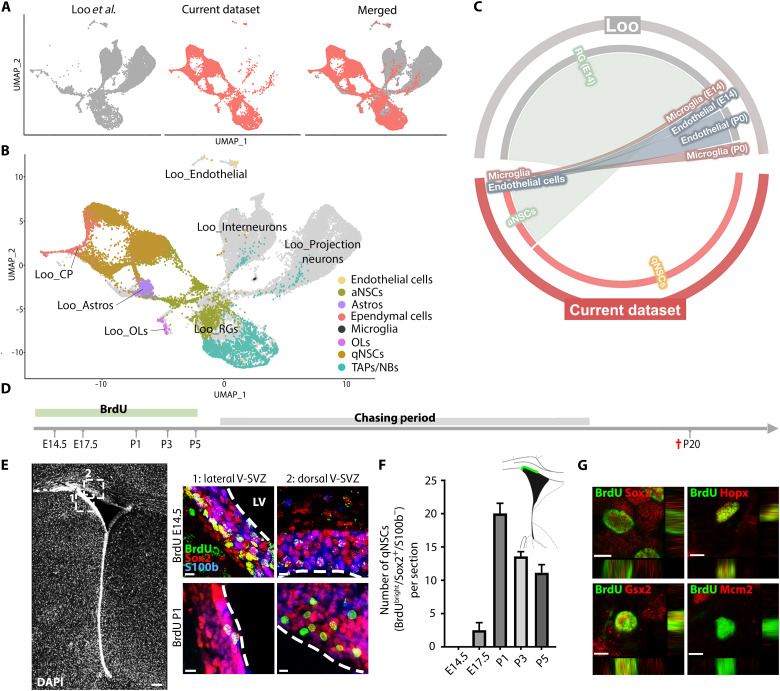
Pallial and subpallial qNSCs are produced postnatally within the dorsal V-SVZ. (**A**) Integration of current P12 dataset (pink) with previously published dataset of E14.5 and P0 pallium (GSE123335; gray). (**B**) Annotated UMAP depicting the identity of both dataset cell types. Only clusters from the current dataset are colored. See legend for cluster identity (CP, choroid rlexus; RG, radial glia). (**C**) Circos plot representing transcriptional correlation between selected cell types of both datasets. Clusters showing transcriptional correlation are relied by line that intensity reflects degree of similarity. (**D**) Scheme illustrating the timeline of the label retaining protocol. BrdU was injected at a selected embryonic or postnatal time, and animals were euthanized at P20. qNSCs are defined on the basis of bright BrdU/Sox2 expression and S100β exclusion. (**E**) Overview showing the location of lateral (1) and dorsal (2) V-SVZ photomicrographs. qNSCs are observed within the lateral V-SVZ only when BrdU is injected at embryonic times (here, E14.5). In contrast, qNSCs are observed within the dorsal V-SVZ when BrdU is injected at postnatal times (here, P1). (**F**) qNSC quantifications showing BrdU^+^/Sox2^+^/S100β^−^ cells per sections into the dorsal V-SVZ following BrdU injection at embryonic or postnatal time points. (**G**) Illustration showing that label-retaining cells into the dorsal V-SVZ coexpressed Sox2 and Hopx or Gsx2 but do not express the cell cycle maker Mcm2. Scale bars, 100 μm (overview, E), 10 μm (insets, E), and 5 μm (G). LV, lateral ventricle.

Both the velocity analysis (Fig. 1G; see also velocity analysis of separated pallial and subpallial lineages in fig. S4, D to F) and the virtual absence of pallial qNSCs in our P2 dataset (Fig. 2Q) suggest their postnatal generation within the dorsal V-SVZ. To confirm this finding, we first integrated our datasets with those of E14.5 and P0 pallial cells (Fig. 3A) (*18*). Differentiated mature cells present in both datasets (i.e., microglial cells, endothelial cells, Astros, and oligodendrocytes) overlapped extensively, validating the robustness of the integration (Fig. 3B). Furthermore, substantial overlaps were observed for aNSCs/TAPs with progenitor/cycling cell populations, as well as for a fraction of TAPs/NBs and cortical neurons, revealing strong transcriptional similarities among both datasets. TAPs/NBs separated in two trajectories corresponding to cortical inhibitory and excitatory neurons, with these cells being rather rare in our datasets because of restriction of the microdissection to the V-SVZ (fig. S4, G and H). In sharp contrast to the extensive overlap observed for all clusters, qNSCs were only observed within our P12 and P22 datasets, supporting their postnatal emergence (Fig. 3B). This was further supported by a ClusterMap analysis, highlighting notable similarities in gene expression between all aforementioned cell types, while on the other hand, qNSCs present in our datasets were entirely segregated from cell types present within the pallium at E14.5 or P0 (Fig. 3C). To confirm the postnatal generation of qNSCs within the dorsal V-SVZ, we used a label-retaining protocol (Fig. 3D). At P20, label-retaining cells expressing bright 5-bromo-2′-deoxyuridine (BrdU) and Sox2, but not the ependymal marker S100β, were never observed following BrdU pulse at E14.5 and were rare following an E17.5 pulse. In contrast, they were frequent following BrdU pulses at postnatal times (i.e., P1, P3 and P5), thereby confirming the postnatal appearance of qNSCs within the dorsal V-SVZ (Fig. 3, E and F). In sharp contrast, we confirmed the presence of qNSCs within the lateral V-SVZ, following BrdU injection at embryonic but not at postnatal time points (Fig. 3E), in agreement with previous studies (*3*, *4*). We finally confirmed that label-retaining cells located within the dorsal V-SVZ coexpressed Sox2, as well as Hopx or Gsx2, and were not positive for the cell cycle maker Mcm2 (Fig. 3G).

**Fig. 4.**
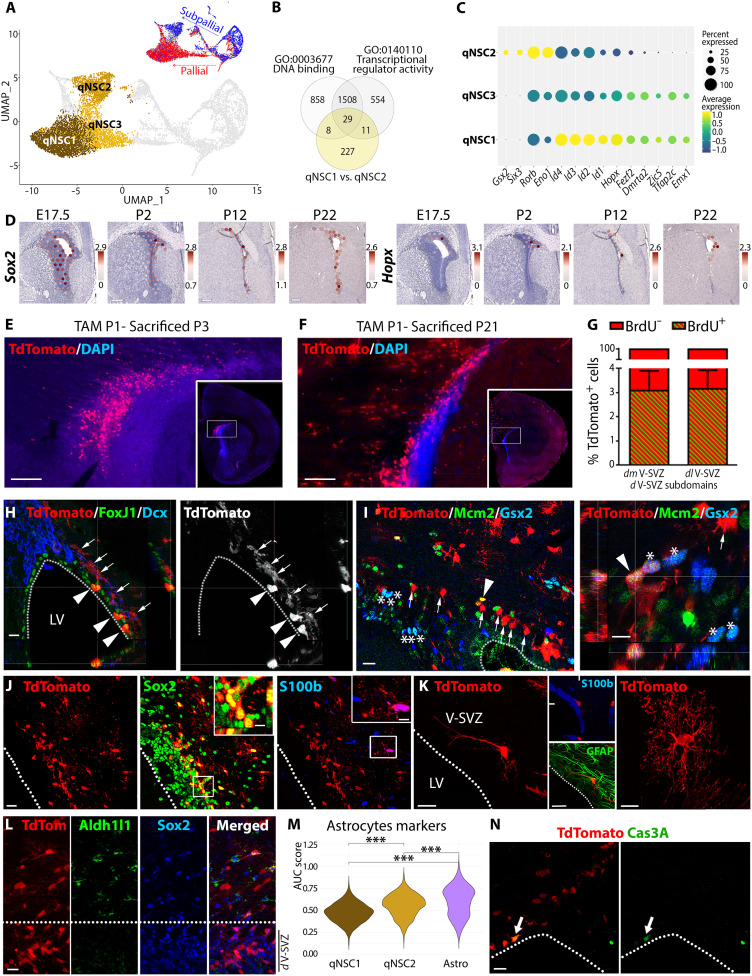
Pallial and subpallial qNSCs are distinguished by Hopx expression. (**A**) UMAP plots highlighting the three qNSC subclusters and the pallial and subpallial trajectories. (**B**) Venn diagram representing differentially expressed genes (DEGs) between qNSC1 and qNSC2 subclusters and identifying TFs/transcription regulators (TFs/TRs) among them. (**C**) Dot plot analysis revealing that some TFs/TRs are enriched or exclusive to a specific cluster. (**D**) Spatial transcriptomics showing expression of *Sox2* and *Hopx* transcripts in the V-SVZ from E17.5 to P22. (**E** and **F**) Fate mapping of Hopx-expressing cells following tamoxifen injection at P1 and euthanasia at P3 (E) or P21 (F). Boxes in overviews indicate the location of the higher-magnification images. (**G**) BrdU injection 2 hours after tamoxifen injection only labels 3% of TdTom^+^ cells at P3 and P21, suggesting quiescence. Subsequent immunohiscochemistry (IHC) was done at P21. (**H**) A small population of TdTom^+^ cells are ECs (Foxj1^+^, green, arrowheads), while others are Foxj1^−^ and do not express the NB marker DCX (arrows). (**I**) Minimal overlap of TdTom^+^ and Gsx2^+^ cells within the dorsal V-SVZ. While Gsx2^+^ and TdTom^+^/Gsx2^+^ cells are frequently Mcm2^+^ (asterisks and arrowheads, respectively), TdTom^+^ cells rarely express this marker (arrows). (**J** and **K**) Most of TdTom^+^ cells within the dorsal V-SVZ express the NSC marker Sox2 (J), show NSCs morphologies (K), and do not express the ependymal/astrocytic marker S100β, while parenchymal TdTom^+^ cells arbor an astrocytic morphology. (**L**) TdTom^+^ cells express Sox2 but not the astrocytic marker Aldh1l1 within the dorsal V-SVZ, unlike in the parenchyma. (**M**) AUC score using best astrocytic markers defined in (*23*). (**N**) Activated caspase 3 (Cas3A) staining reveals rare apoptotic TdTom^+^ cells (arrow) at P21. Scale bars, 250 μm (D) and 10 μm (insets, J). ***P < 0.001.

To identify the transcriptional specificities of pallial and subpallial qNSCs, we next performed differential gene expression analysis between qNSC1 (pallial) and qNSC2 (subpallial) clusters (qNSC3 representing an intermediate cluster toward qNSC1 production as shown by the velocity analysis; Fig. 4A and fig. S5A). Focusing on TFs (Fig. 4B), we confirmed that qNSC1 expresses higher pallial identity markers such as *Emx1*, as well as the transcriptional regulator *Hopx* and the *Id* protein family (Fig. 4C and fig. S5B), which expression within the dorsal V-SVZ was confirmed by spatial transcriptomics (i.e., *Hopx*; Fig. 4D) and by immunostaining (i.e., Id1; fig. S6E). This correlated with an overall reduced transcriptional activity, as reflected by a reduction of 12% in the number of transcripts detected in qNSC1, when compared to qNSC2 (i.e., 3762 versus 4276 detected genes, respectively; fig. S5C). In contrast, qNSC2 showed exclusive expression of subpallial markers including *Gsx2*, as well as higher expression of *Rorb*, *Eno1*, and *Six3*, which has previously been associated with progenitor proliferation (Fig. 4C and fig. S5B) (*19*). The enriched expression of *Hopx* in pallial qNSCs (qNSC1) is of particular interest given its involvement in integrating TGFβ signaling in other systems (*20*), together with its previously reported expression in the postnatal dorsal V-SVZ (*21*) and in adult qNSCs (*22*). We took advantage of *Hopx^CreERT2^* mice to fate map pallial qNSCs at postnatal stages. Following tamoxifen injection at P1, we confirmed the presence of a large TdTom^+^ population of cells within the V-SVZ at P3 that persisted until P21 (Fig. 4, E and F), although their number decreased and became gradually restricted to the rostral-most region of the V-SVZ. At this age, TdTom^+^ cells residing within the V-SVZ were frequently negative for the ependymal marker Foxj1 and for the NB marker Dcx (Fig. 4H), but positive for the progenitor’s marker Sox2 and more rarely to Gsx2 (Fig. 4I). Furthermore, BrdU injection, as well as immunodetection of proliferation markers (Mcm2), confirmed that most fate-mapped cells quickly exited the cell cycle and remained quiescent (Fig. 4, G and I) until P21. TdTom^+^ cells residing within the V-SVZ or adjacent parenchyma showed a notably different morphology and expressed distinct markers (Fig. 4, J to L). Those residing within the V-SVZ showed a radial morphology and expressed glial fibrillary acidic protein (GFAP), but not the ependymal/astrocytic markers S100β and Aldh1l1. In sharp contrast, those residing within the parenchyma showed a typical astrocytic morphology and, accordingly, expressed S100β and Aldh1l1. To further underline the different identities of these two cell populations, we calculated an "astrocytic" area under the curve (AUC) score (*23*) on the basis of expression of the previously published best astrocyte marker genes (*24*). This score was higher for Astros present in our dataset, while it was statistically lower for both qNSC1 and qNSC2 (Fig. 4M and table S7). Last, we performed activated caspase 3 staining, which revealed that only rare TdTom^+^ cells were undergoing apoptosis at P22 (Fig. 4N). Together, these results support the persistence of a large population of pallial qNSCs within the dorsal V-SVZ, which do not acquire an astrocytic phenotype but keep characteristics of NSCs.

### Pallial and subpallial NSCs are defined by distinct levels of quiescence and TGFβ signaling

**Fig. 5.**
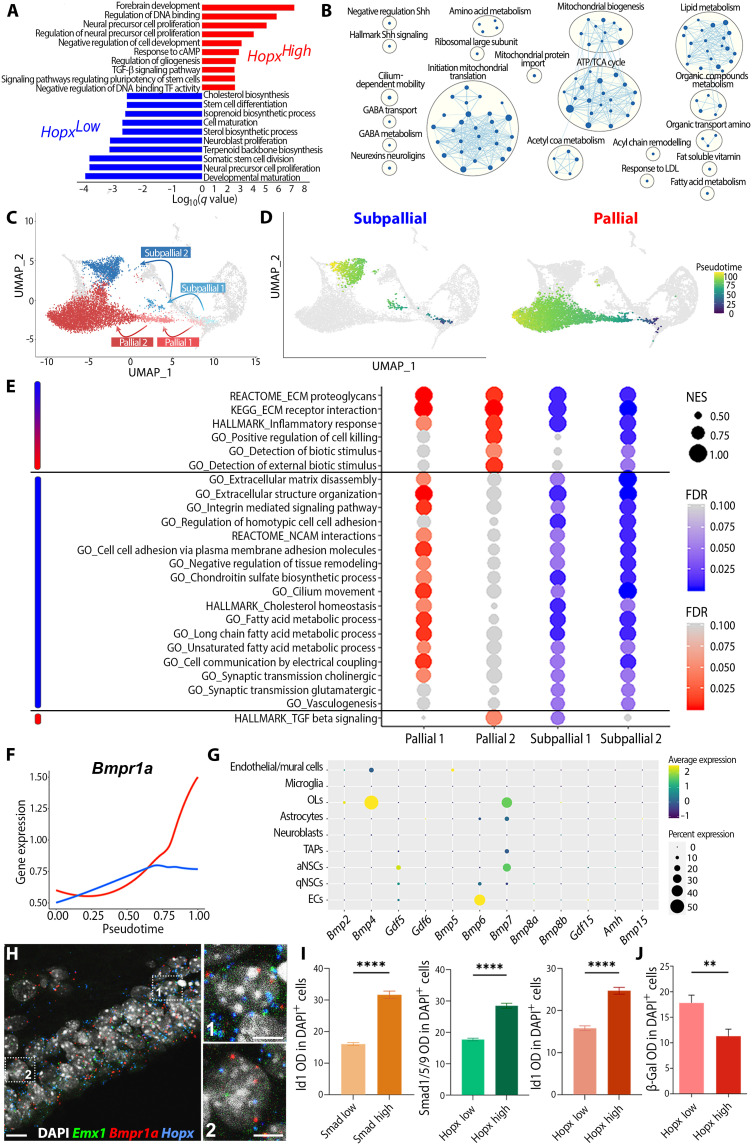
Hopx and TGFβ/BMP signaling define pallial qNSCs quiescence/senescence. (**A**) Bar plot of significantly overrepresented GO terms identified by overrepresentation analysis using genes enriched in *Hopx^High^* (red) or *Hopx^Low^* (blue) cells among qNSCs. See also table S3. (**B**) Hierarchical network of GO terms identified by GSEA using genes enriched in *Hopx^Low^* (blue) cells among qNSCs. Genes enriched in *Hopx^High^* cells did not result in any network. See also table S4. (**C**) UMAP plot illustrating the pallial (red) and subpallial (blue) trajectories, and transition stages resulting in the production of qNSCs. (**D**) Pseudotime highlighting trajectories resulting in the production of qNSCs showing a pallial and subpallial transcriptional signature. (**E**) Identification of key cellular state transitions along both trajectories, and selected GO terms associated with the observed transcriptional changes. Associated GO terms can be grouped in three categories, i.e., showing similar dynamics (top, blue and red), enriched in the “subpallial” trajectory (middle, blue) or in the “pallial” trajectory (bottom, red). See also table S5. (**F**) Pseudotime analysis of *Bmpr1a* expression in both trajectories. (**G**) Dot plot showing the expression of TGFβ family type I receptor ligand transcripts per cell type. (**H**) Expression of *Emx1* (green), *Bmpr1a* (red), and *Hopx* (blue) in the dorsal V-SVZ at P12 using RNAscope. Insets show examples of triple-positive cells. (**I**) Histograms showing higher IHC signal of Id1 in Smad1/5/9^high^-expressing cells and higher expression of Smad1/5/9 and Id1 in Hopx^High^ cells. (**J**) Histogram showing in contrast that β-galactosidase (β-Gal) expression is reduced in Hopx^High^ cells. Scale bars, 10 μm (H) and 5 μm (H, insets). NES, normalized enrichment score; FDR, false discovery rate; OD, optical density.

We next used our transcriptomic datasets to characterize the transcriptional profile of *Hopx* expressing qNSCs. Identification of differentially expressed genes (DEGs) among *Hopx^High^* (mostly pallial/qNSC1 cells)– and *Hopx^Low^* (mostly subpallial/qNSC2 cells; fig. S5D)–expressing cells (731 cells, *Hopx^High^* = *Hopx* > 2.5; 651 cells, *Hopx^Low^* = *Hopx* < 0.4) resulted in the resolving of key ontological categories. *Hopx^High^*-expressed genes were involved in “negative regulation of DNA binding transcription factor activity” (GO:0043433), “negative regulation of cell development” (GO:0010721), and “TGFβ signaling pathway” (GO:0007179). In sharp contrast, *Hopx^Low^* cells expressed genes involved in “stem cell differentiation” (GO:0048863), “cell maturation” (GO:0048469), and various metabolic pathways, suggesting primed activation (Fig. 5A and table S3). This primed activation was further supported by a higher number of detected genes when compared to *Hopx^High^* cells (fig. S5E) and by a Gene Set Enrichment Analysis (GSEA) showing that, while no gene sets were associated with *Hopx^High^* cells, gene sets important for NSCs activation were associated with *Hopx^Low^* cells. In particular, those included “mitochondrial biogenesis,” “lipid metabolism,” and “fatty acid metabolism,” all defining early stages of NSC activation (Fig. 5B and table S4). In addition, markers of deep G_0_ phase (cell cycle inhibitor p27, *Cdkn1b*) were enriched in qNSC1 compared to qNSC2 (fig. S5F), further supporting the state of deep quiescence of those cells (*25*). In contrast, markers of shallow quiescence (e.g., *Cd9*) were enriched in qNSC2, paralleled by the enriched expression of markers of activation (*Six3* and *Egfr*; fig. S5, B and G), as well as of *Vcam1*, a protein acting as an environmental sensor to regulate V-SVZ lineage progression (*26*), and *Cpt1a*, previously shown to regulate NSC activity, through the activation of fatty acid metabolism (*27*). Last, we confirmed deeper quiescence of qNSC1 compared to qNSC2 by calculating a "deep quiescence" AUC score" (*23*) based on markers defined in (*25*) (fig. S5H and table S7).

Pallial (*Hopx^High^*) and subpallial (*Hopx^Low^*) qNSCs emerged through distinct aNSC populations. aNSC1 and aNSC4 appeared to bridge aNSC3 with pallial and subpallial qNSCs, respectively (Fig. 5, C and D; see also Fig. 2A and velocity analysis of pallial and subpallial lineages in fig. S4, D to H). To gain insight on molecular changes defining these two trajectories, we extracted DEGs at each transition stage and performed a GSEA to identify gene sets on the basis of three criteria: (i) similar expression within both trajectories, (ii) predominance in the subpallial trajectory, and (iii) predominance in the pallial trajectory (Fig. 5E and table S5). Only a limited number of gene sets showed a similar induction within both trajectories, among which GOs related to extracellular matrix (ECM) receptor interaction (KEGG:M7098), as well as detection of biotic stimuli (GO:0009595 and GO:0098581), reflecting the increased anchorage of qNSCs within the niche and interaction with their microenvironment (*22*). Similarly, gene sets related to inflammation (Hallmark:M5932) and regulation of cell killing (GO:0031343) were equally up-regulated, implying that at least a fraction of qNSCs may undergo cell death. Genes showing specific up-regulation within the subpallial trajectory were more numerous and often associated with biological functions important for NSCs homeostasis. For example, these included multiple gene sets related to cell adhesion (i.e., « GO:0034110 regulation of homotypic cell-cell adhesion » and « GO:0098742 cell-cell adhesion via plasma-membrane adhesion molecules ») (*22*), cilium biology (GO:0003341 cilium movement) (*28*), fatty acid metabolism (« GO:0006631 fatty acid metabolic process », « GO:0001676 long-chain fatty acid metabolic process », and « GO:0033559 unsaturated fatty acid metabolic process ») (*15*), synaptic transmission (« GO:0007271 synaptic transmission, cholinergic » and « GO:0035249 synaptic transmission, glutamatergic »), and vasculogenesis (GO:0001570 vasculogenesis) (*29*). In sharp contrast, only a few gene sets were specifically enriched in the pallial trajectory. The most noticeable was the “hallmark of TGFβ signaling” (Hallmark:M5896), which was down-regulated in subpallial cells, while constantly increasing within the pallial lineage (Fig. 5E). Because of the convergence of both analysis in identifying TGFβ signaling in pallial qNSCs (i.e., see Fig. 5, A and E), we next explored the temporal pattern of TGFβ receptors’ expression by pseudotime inference (fig. S6, A and B). Our analysis revealed that type I receptor transcripts *Bmpr1a* and *Bmpr1b* showed the highest expression when compared to type II receptors. Among those, we identified *Bmpr1a* as a likely receptor in integrating TGFβ signaling in the pallial lineage, as illustrated by its increasing expression within the pallial qNSCs trajectory, when compared to the subpallial one (Fig. 5F). A comprehensive analysis of TGFβ type I receptor ligands identified *Bmp4*, *Gdf5*, *Bmp6*, and *Bmp7* as putative ligands expressed by OLs, ECs, and qNSCs themselves (Fig. 5G). We confirmed coexpression of *Bmpr1a*, *Emx1*, and *Hopx* within cells of the dorsal V-SVZ at P12, using RNAscope (Fig. 5H and fig. S6C). Furthermore, a pseudotime analysis confirmed the up-regulation of *Bmpr1a* downstream effectors and targets *Smad9* and *Id1/2/3* within the pallial lineage (fig. S6D). Last, we performed immunostaining for Smad1/5/9, as well as for its downstream target Id1 (Fig. 5I and fig. S6E) to confirm the activation of Bmpr1a signaling within the dorsal V-SVZ. Analysis revealed higher levels of expression of these markers in cells expressing high levels of Hopx (Hopx^High^; Fig. 5I). The opposite was observed for BAT-gal signal within the Wnt/β-catenin pathway reporter mice (Fig. 5J), suggesting that Hopx expression promotes Bmpr1a signaling within pallial qNSCs while concomitantly repressing canonical Wnt signaling, as previously observed in other tissues (*20*).

Together, these results highlight the emergence of two qNSCs populations within the postnatal dorsal V-SVZ. Notably, a large cohort expressing pallial genes and persistent TGFβ signaling enters deep quiescence, whereas conversely, a smaller pool expressing subpallial genes remains primed for activation.

### Postnatal induction of deep pallial quiescence is paralleled by a rapid blockade of glutamatergic progenitor/nascent neuron production and differentiation

**Fig. 6.**
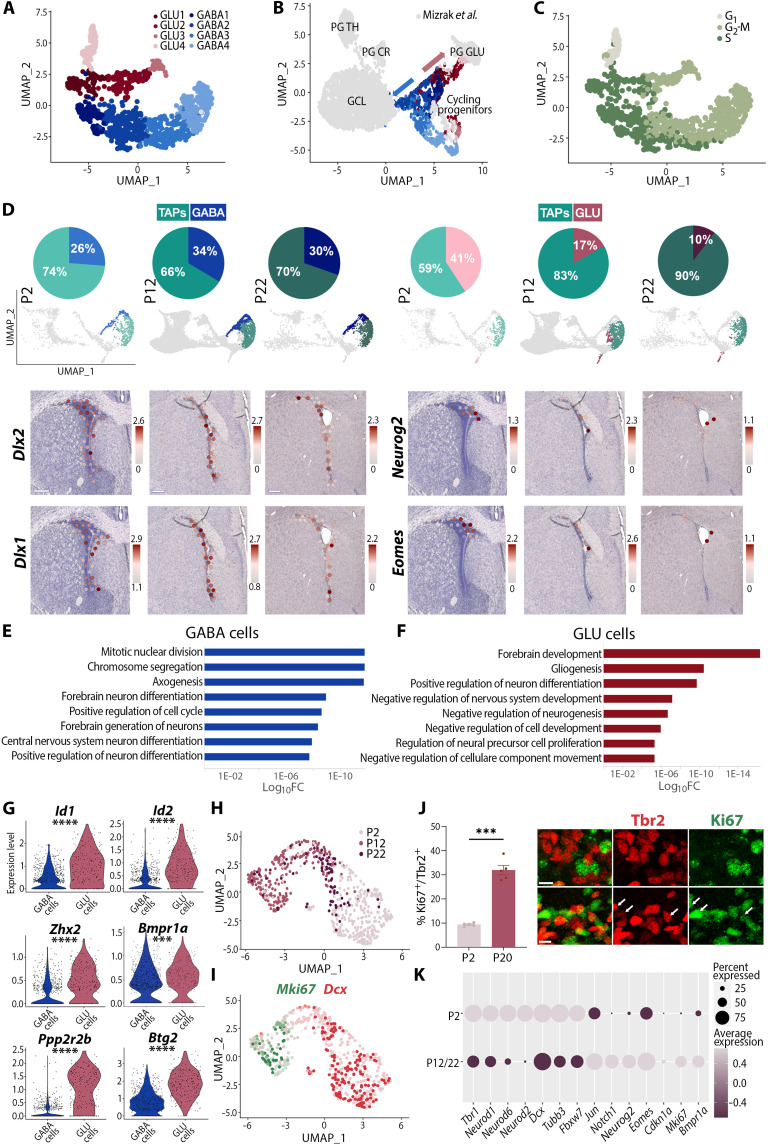
Gradual blockade of pallial lineage neuronal differentiation within the postnatal dorsal V-SVZ. (**A**) UMAP plot with identity of GLU (red gradient) and GABA (blue gradient) TAPs subclusters at P12. (**B**) Annotated UMAP plot showing integration of current P12 dataset (colored) with previously published dataset of postnatally born OB neurons (GSE134918; gray). Two trajectories (arrows) contribute to GABAergic and GLUergic OB neurogenesis. (**C**) Feature plot indicating cycle phase of GLU and GABA cells. (**D**) Pies (and related UMAPs) showing the declining and stable proportion of GLU and GABA cells, respectively, among TAPs at P2, P12, and P22. *Dlx2*, *Dlx1*, *Neurog2*, and *Eomes* expressions revealed by spatial transcriptomics within the V-SVZ at corresponding time points. (**E** and **F**) Bar plots of the selected overrepresented GO terms for genes enriched in GABA cells (E) or GLU cells (F). See also table S6. (**G**) Violin plots illustrating selected genes from representative GO terms overrepresented in GLU cells at P12. (**H** to **I**) UMAP plot of GLU cells at P2, P12, and P22 (H) and feature plot for *Mki67* and *Dcx* (I) illustrating that cell cycle exit and neuronal differentiation decline after P2. (**J**) Histogram and representative images showing that the proportion of cycling Tbr2^+^ cells is lower at P2, than at P20, confirming gradual cell cycle exit blockade. (**K**) Dot plot illustration showing enrichment of representative transcripts enriched in GLU cells at early (P2) or late (P12/P22) time points. Scale color refers to P2 and P22 cells as represented in (H). Scale bars, 250 μm (D) and 10 μm (J). ***P < 0.001, ****P < 0.0001.

To investigate the neuronal output of dorsal NSCs, we isolated cells of the neuronal trajectory (i.e., aNSC3 subclusters 4 and 5 from Fig. 2E, as well as TAPs). Following removal of *Olig1/2*-positive cells, we obtained 1029 cells with a majority (66%) expressing *Pax6* and *Rlbp1*, markers of dorsally born nascent neurons (*14*), while almost none (3 of 1029) expressed markers of ventrally born nascent neurons (*Runx1t1* and *Vax1*). New clustering of these cells followed by lineage identification (see Materials and Methods for details) resulted in the generation of an UMAP plot distinguishing GLU and GABA cells (fig. S7A). Both lineages were subdivided into four subclusters corresponding to distinct steps of their differentiation, which we named GLU1 to GLU4 and GABA1 to GABA4 (Fig. 6A and fig. S7B). Transcriptional integration of these cells with a dataset produced from the postnatal OB (*30*) resulted in a clear overlap of cycling cells, as well as two opposing trajectories toward neuronal populations generated postnatally by the dorsal V-SVZ. The first trajectory corresponded to the GLU lineage in close proximity with periglomerular GLUergic neurons, whose generation persists during postnatal development, albeit at a waned pace (*8*, *9*), whereas the second trajectory corresponded to the GABA lineage projecting toward granular cells and periglomerular CR^+^/TH^+^ interneurons (Fig. 6B). Upon closer inspection of cell cycle marker expression, all GABA subclusters corresponded to cycling cells, suggesting sustained amplification, whereas some GLU cells had exited cell cycle (i.e., GLU4) to become postmitotic (Fig. 6C and fig. S7C).

We next compared the proportion of GABA and GLU cells among TAPs at P2, P12, and P22. While GABA cells remained stable in line with the persistent GABAergic neurogenesis, the proportion of GLU cells rapidly declined (Fig. 6D). This was further illustrated by Visium spatial transcriptomics. While GLU progenitors’ marker expressions (*Neurog2* and *Eomes*) rapidly decreased within the dorsal V-SVZ, expression of GABA progenitors’ markers (*Dlx2* and *Dlx1*) persisted until P22. To investigate the transcriptional correlates of these different dynamics, we identified DEGs between cycling GABA and GLU cells. An overrepresentation analysis revealed key biological processes as differentially regulated between GABA and GLU cells (Fig. 6, E and F). GO categories associated with mitosis [e.g., “mitotic sister chromatid segregation” (GO:0000070), “chromosome segregation” (GO:0007059), “positive regulation of cell cycle” (GO:0045787), “forebrain neuron differentiation” (GO:0021879), and “regulation of axonogenesis” (GO:0050770)] appeared within the top enriched categories in GABA cells (Fig. 6E and table S6). In contrast, several GO categories related to “negative regulation of cell development,” “negative regulation of neurogenesis,” and “negative regulation of nervous system development” were enriched in GLU cells (Fig. 6F and table S6). These results are in line with the sustained expression of transcriptional repressors (i.e., *Id1*, *Id2*, and *Zhx2*), as well as with the higher expression of antiproliferation and negative regulator of cell growth genes (i.e., *Btg2* and *Ppp2r2b*, respectively) in GLU cells (Fig. 6G and fig. S7D).

Last, we subclustered GLU cells from the P2, P12, and P22 datasets to generate a new UMAP. While postmitotic cells expressing *Dcx* but not *Mki67* transcripts were prevalent at P2, their proportion gradually decreased at later time points (Fig. 6, H and I), suggesting a rapid blockade of GLU cell differentiation. This was confirmed by quantification of cycling (i.e., Ki67^+^) cells among Tbr2-expressing (i.e., *Eomes*) GLU cells, which revealed a large increase in their proportion at P20, when compared to P2 (Fig. 6J). Last, to gain insight into the transcriptional correlates of this blockade, we compared the expression of early and late GLU lineage markers at P2 versus P12/P22 (Fig. 6K). This analysis confirmed the enrichment of postmitotic GLU lineage markers at P2 (*Tbr1*, *Neurod1*, *Neurod2*, and *Neurod6*) in line with the higher expression of immature neuronal markers *Tubb3* and *Dcx*. In contrast, early GLU lineage markers *Eomes* and *Neurog2* were enriched at P12/P22, in agreement with a higher expression of the proliferative markers *Cdkn1a* and *Mki67*. *Fbxw7* was down-regulated at this later time point, which, together with the enriched expression of *Jun* and *Notch1*, may participate in the gradual depletion of GLU cells from the dorsal V-SVZ (*31*). Last, *Bmpr1a* appeared to be enriched at P12/P22 in a large fraction of GLU cells.

Together, our findings indicate that a rapid blockade of GLUergic neurogenesis parallels the induction of deep quiescence within pallial NSCs, while GABAergic neurogenesis persists in the postnatal dorsal V-SVZ. Furthermore, persistent high expression of *Bmpr1a* suggests a role for TGFβ/BMP signaling in silencing pallial germinal activity by synchronizing quiescence induction and blockade of neuronal differentiation.

### Manipulation of Bmpr1a signaling modulates dorsal V-SVZ germinal activity

**Fig. 7.**
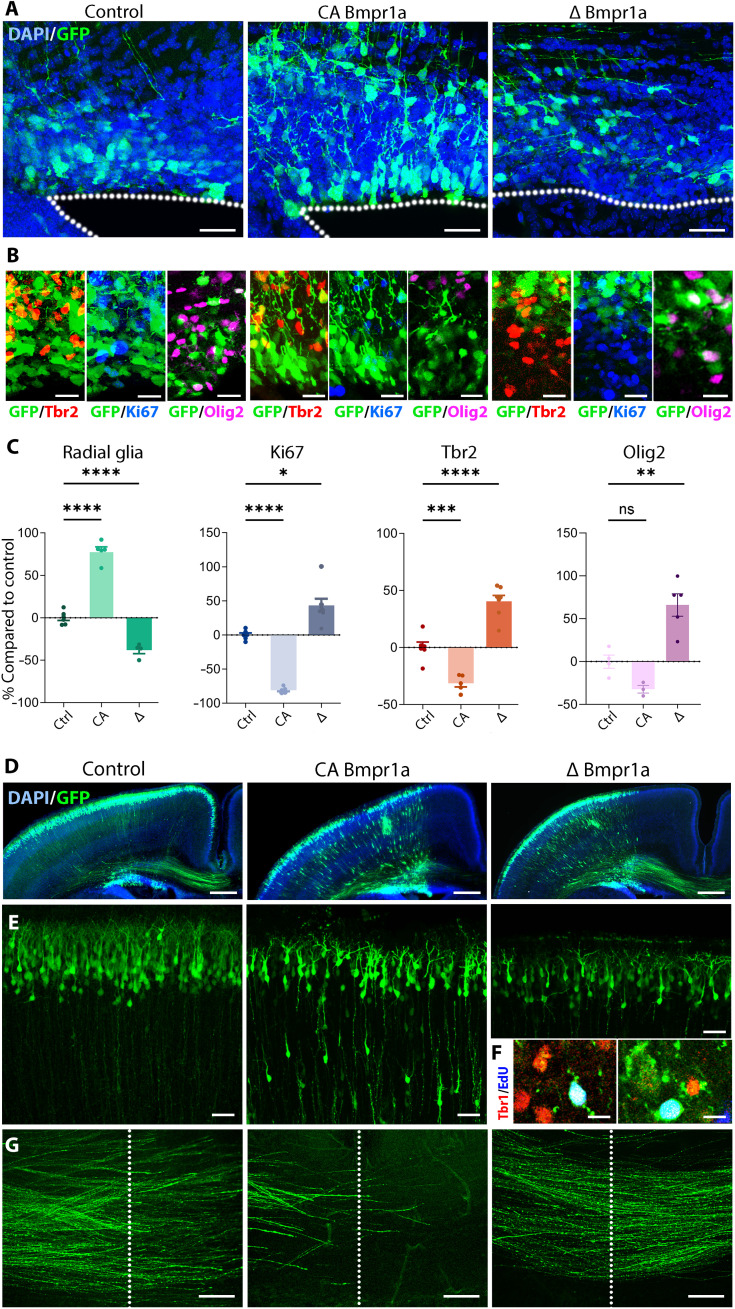
Bmpr1a manipulations modulate postnatal dorsal V-SVZ germinal activity. (**A**) Representative images of the dorsal V-SVZ at P3, electroporated with GFP (control), constitutively active (CA) Bmpr1a, and dominant negative (∆) Bmpr1a plasmids at E16.5. The location of the ventricular lumen is indicated by dotted lines. (**B**) IHC for the pallial progenitor (Tbr2, red), proliferative (Ki67, blue), and oligodendroglia (Olig2, magenta) markers in the three experimental groups. (**C**) Graphs showing the percentage of GFP^+^ electroporated cells showing RG morphology (Ctrl = 6, CA = 5, and ∆ = 4), expression of Ki67 (Ctrl = 6, CA = 5, and ∆ = 7), Tbr2 (Ctrl = 6, CA = 5, and ∆ = 7), or Olig2 (Ctrl = 4, CA = 3, and ∆ = 5). Control values were normalized to 100% to better illustrate the consistent decrease and increase following CA Bmpr1a and ∆ Bmpr1a electroporations, respectively. One-way analysis of variance (ANOVA) was performed. Error bars indicate the SEM. (**D**) Representative overviews of the electroporated animal in the three experimental groups. (**E**) Higher magnification of GFP-positive cells within the P3 cortex. (**F**) EdU injection at P2 reveals the presence of Tbr1^+^/GFP^+^ newborn neurons within the cortex following ∆ Bmpr1a overexpression. (**G**) Axons of GFP-positive cells within the P3 corpus callosum. The midline is indicated by dotted lines. Scale bars, 20 μm (A), 10 μm (B and F), 500 μm (D), and 50 μm (E and G). *P < 0.05, **P < 0.01, ***P < 0.001, ****P < 0.0001, ns, not significant.

To investigate the role of Bmpr1a in shaping postnatal dorsal V-VSZ germinal activity, we performed electroporation of constitutively active, as well as an inactivated, form of Bmpr1a [CA Bmpr1a and ∆ Bmpr1a, respectively (*32*)] within the pallium at late embryonic times (E16.5), a time point corresponding to the neurogenic-to-gliogenic switch. Analysis of the dorsal V-SVZ at P3 revealed profound alterations of the pattern of GFP^+^ cell distribution. While overexpression of CA Bmpr1a resulted in cells keeping a radial glia morphology and remaining in contact with the ventricular lumen, overexpression of ∆ Bmpr1a led to their disappearance (Fig. 7, A and C). In these mice, most GFP^+^ cells were located away from the ventricular surface within the V-SVZ and showed a round morphology, typical of progenitors. These distributions and morphologies were in agreement with a decrease of the number of progenitors expressing Tbr2, Olig2, or Ki67 following CA Bmpr1a electroporation, while their number was consistently increased following ∆ Bmpr1a overexpression (Fig. 7, B and C). Together, these findings confirm an instructive role of TGFβ/BMP signaling on dorsal V-SVZ germinal activity through activation of the Bmpr1a receptor. Hopx gain of function (GoF) resulted in a phenocopy of the results obtained following *CA Bmpr1a* electroporation, supporting a crucial role for Hopx in modulating Bmpr1a signaling to induce the observed effects (fig. S8, A to F).

These effects were not only restricted to the V-SVZ but also affected neuron migration and maturation. Electroporation of CA Bmpr1a resulted in many neurons failing to migrate to the uppermost layers of the cortex (Fig. 7, D and E). A small number of GFP^+^ bipolar cells were also observed within the cortical plate following ∆ Bmpr1a overexpression (visible on Fig. 7D). 5-Ethynyl-2′-deoxyuridine (EdU) administration at P2 confirms that some of these cells were generated at postnatal times and expressed the neuronal marker Tbr1, suggesting an extension of the period of cortical neurogenesis in those animals (Fig. 7F). Last, the maturation of the upper layer neurons was also affected, as reflected by marked differences in their axons. While these later had reached the midline by P3 in control mice, CA Bmpr1a electroporation resulted in a profound decrease of axonal growth (Fig. 7G). This contrasted with neurons produced following ∆ Bmpr1a overexpression, which axons extended farther than in control mice, well into the contralateral hemisphere. Thus, in agreement with an enriched expression of *Bmpr1a* within cells of the pallial lineage, our findings reveal that Bmpr1a signaling affects V-SVZ proliferation as well as the production and maturation of newborn neurons and therefore play a key role in the closure of the period of cortical neurogenesis.

## DISCUSSION

While the closure of GLUergic neurogenesis was believed to mainly rely on a neurogenic-to-gliogenic switch, a recent clonal study revealed that a large number of RGs do not undergo this transition (*5*). Here, we used scRNA-seq and histological analysis of the V-SVZ to investigate at the cellular-level changes occurring in its dorsal-most domain at early postnatal ages. Identification and direct comparison of pallial and subpallial cell lineages within this germinal region reveal transcriptional hallmarks associated with pallial NSC entrance into deep quiescence and a dysregulation of their differentiation potential, which both converge onto a rapid closure of GLUergic neurogenesis following birth. Furthermore, our results highlight a key role for TGFβ/BMP signaling receptor Bmpr1a in silencing pallial germinal activity after birth (fig. S9).

A large population of aNSCs is observed within our dataset, illustrating the sustained germinal activity observed within this region at early postnatal time points (*1*). We could observe an early fate priming of these cells, as illustrated by their clear segregation in clusters expressing TFs known to instruct neurogenesis, astrogenesis, and oligodendrogenesis. Furthermore, our results support the coexistence of both unipotent and bipotent NSCs, as reflected by the coexpression of neurogenic and oligodendrogenic TFs (e.g., *Eomes* and *Olig2*) in some aNSCs and TAPs. These observations are in line with the early priming of adult NSCs (*10*), as well as with recent clonal fate mapping studies within the postnatal V-SVZ (*34*). Thus, while at the population level, NSCs appear to act in a rather homogeneous manner; analysis at the single-cell level reveals an unsuspected level of heterogeneity.

RNA velocity reveals several trajectories emerging from aNSCs, the most noticeable driving them toward quiescence. Thus, although major transcriptional differences between the dorsal and lateral V-SVZ have been shown by previous studies (*7*, *35*), our work highlights important differences in NSC dynamics, particularly in the timing of quiescence induction. While original studies have pointed at an embryonic origin of adult NSCs [i.e., “set-aside” model (*3*, *4*)], our results indicate that this rule does not apply to the dorsal V-SVZ. Both our transcriptomic and histologic analyses reveal an entrance into quiescence at perinatal ages. These conclusions are in line with fate mapping of embryonically born qNSCs in the H2B-GFP mouse following E9.5 induction, which revealed that GFP-retaining cells are observed within the lateral but not the dorsal-most regions of the juvenile V-SVZ (*4*). Similarly, our results confirm that BrdU injection at E14.5 results in the detection of numerous positive cells within the lateral and ventral V-SVZ, while no cells are observed within the dorsal V-SVZ (*3*). Thus, while a population of qNSCs appears to diverge from embryonically active ones within the ganglionic eminence and to persist at postnatal ages in the lateral and ventral V-SVZ; those from the dorsal V-SVZ enter quiescence around birth, in line with a “continuous model” recently proposed within the hippocampus (*36*). Knowing the importance of the tight regulation of qNSCs/aNSCs balance in maintaining germinal activity, it is tempting to speculate that this temporal difference results in distinct states of germinal activity sustainability within the V-SVZ. Thus, while NSCs derived from the subpallium remain germinaly active in producing various OB interneurons throughout life, NSCs derived from the pallium declines rapidly in their capacity to produce GLUergic periglomerular cells (*8*, *9*), and then CR^+^ interneurons (*37*), possibly through their rapid exhaustion or induction of irreversible quiescence.

NSCs expressing pallial or subpallial markers coexist within the postnatal V-SVZ dorsal domain, allowing their direct comparison. Distinct levels of quiescence characterize these two-cell populations. Parallel comparison of pallial and subpallial quiescence trajectories reveals the synchronized and persistent expression in subpallial qNSCs of genes involved in biological functions known to be important for germinal activity maintenance. For instance, genes involved in ECM reorganization and regulation of cell adhesion are observed within subpallial qNSCs, in agreement with the importance of NSC interactions with their microenvironment in regulating quiescence/activation balance (*22*, *38*). This is illustrated by the marked enrichment of *Vcam1* that was shown to act in adult NSCs induction and maintenance (*26*, *39*). Although also observed at the early stages of the pallial qNSCs trajectory, these gene sets appear to gradually vanish from pallial qNSCs, suggesting their gradual loss of anchorage within the ECM and progressive isolation from both the vasculature and cerebrospinal fluid (CSF). This is supported by our observation that most pallial qNSCs (Hopx-derived Sox2^+^/BrdU^−^; see below) can be seen at P21 at some distance from the ventricular lumen and in rostral V-SVZ regions, where the ventricle has collapsed. It is likely that there is a spatial component to this deep quiescence induction, which remains to be fully explored. Hopx expression shows a mediolateral gradient of expression in the dorsal V-SVZ, which is opposing expression of proliferative or neuronal markers (*21*). Furthermore, our spatial transcriptomic data indicate that subpallial markers (i.e., *Gsx2* and/or *Dlx2*) appear to be largely restricted to the lateral-most aspect of the postnatal dorsal V-SVZ, where Shh signaling has been shown to occur following birth (*40*). Deeper quiescence is further supported by multiple observations, including a reduced level of *P27* (*Cdkn1b*) and *Egfr* expression, which defines acquisition of a primed GO state (*25*) and entrance in proliferation (*41*), respectively. It remains to be determined whether this deep quiescence is reversible, such as, for example, following early life injuries to produce new cortical neurons. Such plasticity of fate has been observed within the cerebellum, where Hopx^+^ progenitors produce both inhibitory and excitatory cerebellar neurons, in addition to their main gliogenic output, following neonatal X-irradiation (*42*).

Our results highlight *Hopx* as a marker of pallial qNSCs. *Hopx* was identified as a quiescent stem cell marker in intestines (*43*) and hematopoietic stem cells (*44*) in mice, as well as in dentate gyrus NSCs (*36*), a region where postnatal GLUergic neurogenesis persists. The restricted *Hopx* expression within pallial NSCs allowed us to fate map those cells at early postnatal stages (P1). At this early stage, a large proportion of *Hopx*^+^ cells are nonproliferative as revealed by the absence of BrdU incorporation. Furthermore, at least a portion of those cells persist within the dorsal V-SVZ for at least 3 weeks after birth (our observations) and probably later [see, for example, (*14*)]. *Hopx* transcript remains detectable within adult qNSCs, where it shows a >33-fold enrichment when compared to aNSCs (*22*). Fate mapping of *Hopx*-expressing cells at 2 months results in no neurons being labeled in the OB (*45*), further supporting deep, irreversible quiescence. It is unclear whether *Hopx* contributes directly to the induction of deep quiescence. A comparative GSEA analysis of *Hopx^High^* and *Hopx^Low^* qNSCs fails to reveal significant gene set induction in *Hopx^High^* cells, while its low expression correlates with priming for activation, as reflected by induction of ribosomal and mitochondrial biogenesis transcripts. Thus, while *Hopx* knockout disrupts stemness and quiescence of hematopoietic stem cells in mice (*44*), its role is likely to be more complex within the postnatal V-SVZ. Previous experiments aimed at investigating the consequences of Hopx GoF and LoF (loss of function) within the postnatal V-SVZ failed to demonstrate a marked effect on germinal activity, although induction of quiescence was not investigated (*21*).

The molecular mechanisms underlying Hopx^+^ NSCs quiescence appear to be at least partially related to regulation of BMP versus Wnt signaling. Hopx has been shown to modulate primitive hematopoiesis by inhibition of Wnt signaling (*46*), and modulation of BMP/Wnt signaling by Hopx has been shown to occur in the developing heart (*20*), as well as during endothelial development and primary hematopoiesis (*46*). Our measurements of BMP/Wnt signaling by immunodetection of Smad1/5/9 and β-galactosidase in the BAT-gal mice support a similar integration of the two pathways by Hopx within the postnatal dorsal V-SVZ. Our transcriptional results suggest a persistent activation of BMP signaling in pallial qNSCs. Furthermore, the transcriptional profile of pallial qNSC resembles those of NSCs exposed to BMP4 in vitro, which, in the absence of fibroblast growth factor 2, result in induction of deep quiescence (*25*, *47*). BMPs belong to the superfamily of TGFβ cytokines and exert a plethora of effects in the nervous system, ranging from dorsoventral patterning to induction of the neurogenesis-to-gliogenesis switch (*48*). Thus, BMP signaling may play a pleiotropic role in pallial lineage germinal activity closure by acting in both astrogenesis in concert with JAK-STAT (Janus kinase–signal transducer and activator of transcription) signaling (*49*) and induction of deep quiescence for cells of the pallial lineage. Our observation that stemness transcripts are retained, while astrocytic marker expression (i.e., Aldl1h1 and S100β) remains absent at P21 in the Hopx progeny within the V-SVZ, suggests that BMP signals do not drive terminal Astro differentiation but rather impose quiescence in at least a fraction of pallial NSCs (*25*). Thus, although Hopx expression has been associated with parenchymal astrogenesis in both mice (*21*) and monkeys (*50*), the fate of Hopx^+^ cells within the V-SVZ appears to be distinct as supported by a reduced score of qNSCs when compared to Astros, when using astrocytic enriched transcripts (*24*). This is in line with a role of TGFβ/BMP signaling in regulating the temporal identity and potency of NSCs in other regions of the CNS, such as the hindbrain (*51*) and the retina (*52*). Identification of TGFβ family members and receptors within the dorsal V-SVZ niche suggests that BMP4 and BMP5 are involved, which are secreted by oligodendrocyte precursor cells/newly formed OLs and NSCs themselves, respectively (*7*). Furthermore, enrichment for *Bmpr1a* expression is observed in pallial qNSCs. Bmpr1a is responsible for triggering the canonical BMP pathway by phosphorylating the SMAD1/5/9 proteins, which translocation is associated with the up-regulation of target genes, such as Id1-4 (*47*), all of which show higher expression in pallial qNSCs, as shown here at the transcriptional and protein level, and are known to block the action of prodifferentiation basic helix-loop-helix TFs (*33*). This elevated expression of several inhibitors of transcription correlates with a mild reduction in their transcriptional activity, possibly by the recruitment of class I histone deacetylases as observed in other tissues (*53*). This is in line with our GoF and LoF experiments, which demonstrate that manipulating *Bmpr1a* results in profound changes in the germinal activity of the dorsal V-SVZ. While activation of Bmpr1a results in a persistence of RG cells at postnatal times, its inactivation has the opposite effect by releasing proliferating and GLUergic (i.e., Tbr2^+^) progenitors’ production. These results are in line with previous work showing a role of Lrp2 in regulating BMP signaling within the V-SVZ to allow sustained neurogenesis (*12*, *54*).

Although the entrance of pallial NSCs in a state of deep quiescence may solely explain the silencing of pallial germinal activity early after birth, other mechanisms appear to participate in the rapid decline of postnatal GLUergic neurogenesis. For instance, it was recently proposed that some pallial progenitors acquire subpallial traits at postnatal time points (*17*, *55*), which correlates with the demonstration of a subpopulation of OB GABAergic neurons deriving from Emx1 progenitors at postnatal ages (*37*) under the influence of Shh signaling, which activity rises in the dorsal V-SVZ at postnatal stages (*40*). These findings are in line with our observation of a substantial number of dual cells, coexpressing markers of both the pallial and subpallial lineages, within our dataset, as well as by our fate mapping of E15.5 pallial NSCs. Our results, however, show that this “reprogramming” of pallial NSCs is largely incomplete, with a subpopulation of TAPs presenting a clear GLUergic neuronal identity observable by scRNA-seq until at least P22, in line with our previous histological and fate mapping studies (*6*, *56*, *57*). Their transcriptional comparison with surrounding GABAergic TAPs reveals a reduced proliferative capacity, as well as a failure of expression of genes necessary for tangential migration toward the OB. Furthermore, their transcriptional analysis at distinct postnatal time (i.e., P2 to P22) supports their rapid failure to undergo efficient differentiation in line with their persistent expression of Id proteins and *Bmpr1a*. These observations are in line with observations made following *Bmpr1a* GoF and LoF. Thus, while activation of Bmpr1a at late embryonic time points results in many neurons failing to migrate and mature, its inactivation results in a more rapid development of interhemispheric axonal projection, as well as in a prolongation of the period of cortical neurogenesis as supported by the observation of EdU^+^/Tbr1^+^ neurons produced at postnatal stages.

Together, our results represent the first characterization of the postnatal dorsal V-SVZ by scRNA-seq. By allowing a direct comparison of pallial and subpallial cell lineages, our work elucidates events that ultimately result in the rapid closure of the period of glutamategic neurogenesis while GABAergic neurogenesis persists throughout life. In particular, the high *Bmpr1a* expression within cells of the pallial lineage appears to play a key role in synchronizing quiescence induction and silencing of neuronal differentiation to rapidly silence pallial germinal activity after birth.

## MATERIALS AND METHODS

The code for the analysis of scRNA-seq data is available at https://github.com/OlivierRaineteauSBRI/scRNASeq.

### Animals and ethics

Mice used in this study were OF1 wild-type (Charles River Laboratories, France), *Hopx^CreERT2^* (*43*), and *Gad1^tm1Tama^* (also called *GAD67^GFP^*) (*58*) transgenic mice, as well as Wnt/β-catenin pathway reporter mice [BAT-gal (*59*)]. In *Hopx^CreERT2^* animals, subcutaneous tamoxifen (Sigma-Aldrich) administration (1 mg per pup) was performed at P1 (i.e., 24 hours following birth). All animal experiments were performed in accordance with European requirements 2010/63/UE that had been approved by the Animal Care and Use Committee CELYNE (APAFIS, nos.187 and 188). Mice were group-housed, with food and water ad libitum, under 12-hour light–12-hour dark cycle conditions.

### Tissue preparation for scRNA-seq

#### Single-cell isolation

The dorsal V-SVZ from P12 mice of two independent experiments, as well as of P2 and P22 mice, were dissected. Two mice were microdissected and pooled by time points. We aimed at selecting only male mice; however, post hoc analysis based on *Xist* detection indicated that a female was included in the P2 time point. Nonetheless, a careful comparison for cell type composition and gene expression levels indicated no change between male and female isolated cells. During all stages of the dissociation protocol, the tissue was kept in artificial CSF solution containing 125 mM NaCl, 2.5 mM KCl, 1.25 mM NaH_2_PO_4_, 26 mM NaHCO_3_, 17 mM glucose, 1.25 mM CaCl_2_, and 1 MgCl_2_. The small dissected tissues were incubated in papain during 20 min at 37°C. Following enzymatic digestion, cells were centrifuged for 5 min, and the pellet was resuspended with deoxyribonuclease/ovomucoid inhibitor according to the manufacturer’s protocol (Worthington). Cells were then centrifuged and resuspended on ice in Leibovitz L-15 medium supplemented with 0.1% of bovine serum albumin. The cell suspension was finally filtered through a 70-μm cell strainer to remove aggregated cells.

#### Fluorescence-activated cell sorting

Viable cells (DAPI^−^ and Hi Draq5^+^) were then sorted using a BD FACSAria sorter. A total of 30,000 cells were collected per replicate in ice-cold phosphate-buffered saline.

### Single-cell RNA sequencing

Cell suspension (300 cells/μl) was added to the 10x Genomics Chromium Single-Cell Controller (10x Genomics) to achieve 7000 encapsulated cells per replicate. The next steps for cDNA synthesis and library preparation were done following the manufacturer’s instructions (chemistry V3). Libraries have been sequenced independently using the Novaseq 6000 platform (Illumina) to target 100,000 reads per cell. Cell Ranger version 3.0.1 was used to align reads on the mouse reference genome GRCm38 mm10 and to produce the count matrix.

### Analysis of scRNA-seq data

#### Quality control and filtering

We filtered 11,298 cells (P12 dataset), 4037 cells (P2 dataset), and 4254 cells (P22 dataset) on the basis of two quality control criteria: the number of genes per cell and the fraction of counts for mitochondrial genes per cell. Cells with <2500 genes or >7500 genes and with >10% mitochondrial gene fraction were removed.

#### Clustering analysis

We first focused our analysis on the P12 replicates. Filtering and data analysis were performed using the R package Seurat (version 3.1) (*60*). First, genes expressed in less than three cells were removed in each dataset. Gene expression was normalized using the standard Seurat workflow, and the 2000 most variable genes were identified. Then, the two batches were integrated using the integration function of Seurat, and anchor genes were scaled without any type of regression and used for principal components analysis (PCA) at 50 dimensions. We then performed preliminary clustering with permissive parameters to identify and remove low-quality clusters. We only removed one cluster of 19 cells that expressed fewer genes compared to the average. The remaining cells were subjected to second-level clustering (20 PCs; resolution = 0.5), yielding 15 final clusters (full_identity), and merged into 9 clusters (simplified_identity). The clusters were visualized in two dimensions using the RunUMAP() function (minimum distance = 0.3; n_neighbors = 30 L; UMAP.method = ‘UMAP-learn’; metric = ‘correlation’). We then performed differential expression analysis and annotated clusters as detailed below. Identification of cell clusters within the P2 and P22 datasets was achieved on the basis of P12 cell types using the MapQuery() function. For quantitative analysis of P2, P12, and P22 datasets (e.g., Fig. 2Q), NBs and OLs were excluded to avoid possible influences from the microdissection that may result in the inclusion of some root mean square or overlying corpus callosum.

#### Subclustering

For subclustering of aNSCs, corresponding to 1514 cells, a resolution of 0.5 was used, leading to the appearance of four subclusters. For subclustering of aNSC3, representing 569 cells, a resolution of 2 was used. Last, for subclustering of the neuronal trajectories, corresponding to 1029 cells, a resolution of 0.75 was used.

#### Transcriptional criteria for cell type identification

We defined the various cell types based on marker combinations (normalized logged counts): qNSCs (*Slc1a3* > 1, *Prom1* > 0.6, *Egfr* < 0.1, and *Foxj1* < 0.1), aNSCs (*Egfr* > 1, *Ascl1* > 1, *Dlx1* < 0.01, and *Dlx2* < 0.01), TAPs (*Egfr* > 1, *Ascl1* > 1, *Dlx1* > 0.01, *Dlx2* > 0.01, *Sp8* < 0.5, *Gad1* < 0.5, and *Gad2* < 0.5), NBs (*Dcx* > 1, *Cd24a* > 0.5, and *Nrxn3* > 0.5), OLs (*Pdgfra* > 0.5 and *Sox10* > 0.5), and Astros (*S100b* > 0.5 and *Aqp4* > 2). Pallial cells were defined as follows: (*Emx1* > 0.25|*Neurod1* > 0.25|*Neurod6* > 0.25|*Neurog2* > 0.25|*Tbr1* > 0.25) and (*Gsx2* < 0.25, *Dlx6* < 0.25, *Dlx5* < 0.25, *Sp9* < 0.25, *Dlx2* < 0.25, and *Gad1* < 0.25). Subpallial cells were defined as follows: (*Gsx2* > 0.25|*Dlx6* > 0.25|*Dlx5* > 0.25|*Sp9* > 0.25|*Dlx2* > 0.25|*Gad1* > 0.25) and (*Emx1* < 0.25, *Neurod1* < 0.25, *Neurod6* < 0.25, *Neurog2* < 0.25, and *Tbr1* < 0.25).

#### RNA velocity

We predicted the direction of transcriptional changes using the RNA velocity framework, which estimates the gene expression dynamics from exonic and intronic expression. We first annotated spliced, unspliced, and ambiguous reads using the run10x command (velocyto.py). RNA velocities were then predicted using the R package scVelo (*16*) on the basis of the 2000 most variable genes, 30 PCs, and 30 neighbors. Estimated RNA velocities are represented on the UMAP with streamlines (scv.pl.velocity_embedding_stream). The direction of the arrows indicates the estimated future state of the current cells. Long arrows correspond to large gene expression changes. We determined RNA velocity using stochastic and dynamical models.

#### Data visualization

To display the gene expression, the preprocessed unique molecular identifier (UMI) matrix was normalized with the function library.size.normalization of the R package Magic (*61*). The dropout corrected data were displayed on the Seurat feature plots with the viridis scale colors. Otherwise, the gray-red scaled feature plots illustrate the original noncorrected UMI matrix.

#### Pseudotime

Both pallial and subpallial quiescent cell lineages were isolated, and the R package slingshot was used to calculate the pseudotime values using the cluster aNSC3 as the root of the trajectory (Fig. 5D).

#### Differential expression analysis

To identify enriched genes, we performed a nonparametric Wilcoxon rank sum test using the FindMarkers() function from Seurat. *P* value adjustment is performed using Bonferroni correction based on the total number of genes in the datasets. Genes with a *P* value of <0.05, at least 0.25 average fold change (log scale), and at least 25% cluster-specific detection (percentage of cells expressing a particular gene in a cluster) were defined as enriched genes for each cluster.

#### Cell cycle assignment

For each cell, we computed a score based on its expression of G_2_-M and S phase markers; cells that express neither of these markers are likely not cycling or are in G_0_-G_1_ phase. The scoring strategy is described in (*62*). We used Seurat’s implementation of this strategy in the CellCycleScoring() function.

#### Identification of DEGs

Identification of DEGs was based on the following criteria: min.pct = 0.1, logfc.threshold = 0.25.

#### Identification of TFs and transcriptional regulators

DEGs were compared to gene sets “DNA Binding” (GO:0003677) and “Transcriptional activity” (GO:0140110).

#### Gene set enrichment

Overrepresentation analyses were performed using the R package “Cluster Profiler” (*63*). DEGs were extracted using the following criteria: Logfc.threshold = 0.25, min.pct = 0.1.

GSEA analyses (*64*) were performed using the GSEA software v4.1.0 [Built 27] from the board institute [GSEA (gsea-msigdb.org)] using the following curated gene set databases: Hallmark “hall.v7.3.symbols.gmt,” Kyoto Encyclopedia of Genes and Genomes (KEGG) “c2.cp.kegg.v7.3.symbols.gmt,” Reactome “c2.cp.reactome.v7.3. symbols.gmt,” and GO “c5.go.bp.v7.3.symbols.gmt.” Obtained results were exported in Cytoscape 3.8.1 for visualization and analysis (www.cytoscape.org).

#### Combined gene expression score

We used the R package AUCell to calculate a composite area under the curve (AUC) “astrocytic" AUC score” (Fig. 4M) using the following genes: *Fam107a*, *Cbs*, *Mlc1*, *Ntsr2*, *Clu*, *Ppp1r3g*, *Gm11627*, *Mfge8*, *Cldn10*, and *S1pr1* (*24*). For calculation of a “deep quiescence" AUC score (fig. S5H), the following genes were used: *Notch2*, *Foxg1*, *Prom1*, *Id1*, *Foxo3*, *Sox2*, *Sox9*, *Pdgfra*, *Nr2e1*, *Ascl1*, and *Nfix* (*25*).

#### ClusterMap analysis

A correlative analysis was performed using the R package “ClusterMap.” It provides a way for automatically and unbiasedly matching subgroups based on a list of marker genes detected in the analysis. ClusterMap can generate circular graphs, called circos plots, between several datasets. It identifies the similarity of clusters, based on transcriptional similarities, and represents them as an arc linking one or more clusters together. First, the gene list for each subcluster in each sample is used to match the subcluster between samples. The file containing the gene list can be the direct output of the Seurat package’s “FindAllMarkers” function. The arcs in the circos plot indicate the subgroups’ relationships. The width of the gray or pink sectors represents the proportion of cells in each sample. Last, the degree of similarity between the matched groups is shown by the transparency of the hue of the arcs. Similarity is a measure of the percentage of overlapping genes between groups (*65*).

#### Integration of multiple datasets

Integration was done by identification of cells that are in the same biological state (anchors) using the function FindIntegrationAnchors() of the Seurat R package. The function IntegrateData() was then applied with the previous anchor set to perform dataset integration using the rpca (Reciprocal PCA) approach. The following datasets were used for integration: GSE123335 dataset corresponding to embryonic (E14.5) and neonatal (P0) pallium and overlying cortex (*18*), as well as GSE134918 dataset corresponding to P56 and P70 V-SVZ and OB (*30*).

#### Label-retaining protocol

A single injection of BrdU (50 mg/kg, intraperitoneally) was made in pregnant mice at E14.5, E17.5, or in newborn mice (P1, P3, or P5). Mice were euthanized at P20.

### Histology and immunostainings

Mice were euthanized by an intraperitoneal overdose of pentobarbital followed by perfusion with Ringer’s lactate solution and 4% paraformaldehyde (PFA) dissolved in 0.1 M phosphate buffer (PB; pH 7.4). Brains were removed and postfixed for 12 to 48 hours at 4°C in 4% PFA and sectioned in 50-μm-thick coronal serial sections. When necessary, antigen retrieval was performed for 20 min in citrate buffer (pH 6) at 80°C, then cooled for 20 min at room temperature, and washed in 0.1 M PB. Immunostainings were performed as previously described (*6*, *21*). For double staining in which primary antibodies were produced in the same animal (i.e., Smad 1/5/9 and Id1), a more complex protocol was used (*66*), as previously reported (*67*).

Blocking was done in Tris-NaCl-blocking (TNB) buffer (0.1 M PB, 0.05% casein, 0.25% bovine serum albumin, and 0.25% TopBlock) with 0.4% Triton X-100 (TNB-Tx). Sections were incubated overnight at 4°C with gentle shaking the following primary antibodies in TNB-Tx. The following primary antibodies were used for immunohistochemical procedures: rabbit anti-Hopx (1:400; Santa Cruz Biotechnology, sc-30216), guinea pig anti-Dlx2 (1:5000; gift of K. Yoshikawa), mouse anti-Hopx (1:400; Santa Cruz Biotechnology, sc-398703), goat anti-DCX (1:500; Santa Cruz Biotechnology, sc-8066), mouse anti-GFAP (1:500; Millipore, MAB3402), goat anti-Mcm2 (1:300; Santa Cruz Biotechnology, sc-9839), mouse anti-Sox2 (1:1000; R&D Systems), guinea pig anti-S100β (1:2000; synaptic system), rat anti-Tbr2 (1:1000; Invitrogen, 14-48-75-82), rabbit anti-Tbr2 (1:1500; Millipore, AB2283), rabbit anti-Gsx2 (1:2000; Millipore, ABN162), rabbit anti-S100β (1:5000; Swant), rat anti-BrdU (1:1000; Abcam, ab6326), mouse anti-BrdU (G3G4) (1:1000; DSHB, 7/5118), rabbit anti-Tbr1 (1:500; Abcam, AB31940), chicken anti-GFP (1/1000; AVES), rabbit anti-Smad1/5/9 (1/250; Cell Signaling Technology, 13820S), rabbit anti-Id1 (1/500; biotech, BCH-1#37), rabbit anti-Aldh1l1 (1/500; Abcam, ab28897), chicken anti–β-galactosidase (1/2000; Abcam, ab9361), rabbit anti-activated caspase 3A (Cas3A; 1/1000; Millipore, Ab3623), rabbit anti–red fluorescent protein (1/500; MBL, PM005), and goat anti-mCherry (1/200; Sicgen, AB0081). Following extensive washing in 0.1 M PB with 0.4% Triton X-100 (PB-Tx), sections were incubated with appropriate secondary antibodies conjugated with Alexa Fluor 488, 555, or 647 (1:500; Life Technologies) for 2 hours at room temperature. Sections were washed and counterstained with 4′,6-diamidino-2-phenylindole (DAPI) (1:5000; Life Technologies, D1306) and then mounted with Fluoromount-G (Clinisciences, 0100-01).

### RNAscope

A mouse brain (P12) was paraffined and serially sectioned using a Leica microtome at 5-μm thickness on TOMO slides. Sections were incubated at 60°C for 1 hour and then deparaffinized for 10 min with RNAscope hydrogen peroxide at room temperature and washed in distilled water. Samples were then treated with 1× target retrieval reagent for 15 min at 99°C. Next, samples were rinsed in distilled water for 15 s followed by a bath of ethanol 100% for 3 min at room temperature. Last, samples were surrounded by a hydrophobic barrier (Dako pen) and were allowed to dry overnight at room temperature. The day after, samples were covered with RNAscope Protease Plus for 30 min at 40°C and washed in distilled water. Hybridization of probes and amplification solutions was performed using the RNAscope Multiplex Fluorescent Reagent Kit V2 (catalog no. 323100) according to the manufacturer’s instructions. Probe combinations used were the following: Mm-Emx1 (#312261), Mm-Gsx2-C2 (#554261-C2), Mm-Hopx-C3 (#405161-C3), Mm-Emx1 (#312261), Mm-Bmpr1a-C2 (#312421-C2), and Mm-Hopx-C3 (#405161-C3). Samples were incubated with the probes mixed or with positive and negative control probes for 2 hours at 40°C. Samples were then washed with 1× buffer for 2 min at room temperature and incubated with RNAscope Multiplex FL V2 Amplification buffers (AMP1 to AMP3) for 30 min at 40°C and then washed in 1× buffer. This was followed by incubation with RNAscope Multiplex FL V2 HRP-C1 for 15 min at 40°C and then Opal 520 for 30 min at 40°C at 1:1500. Samples were incubated with a horseradish peroxidase (HRP) blocker RNAscope Multiplex FL V2 for 15 min at 40°C. The same procedure was done with Opal 570 and 690 for C2 and C3 probes. Last, samples were counterstained with DAPI for 30 s at room temperature and coverslipped in Fluoromount-G medium (Clinisciences, 0100-01). Parameters for image acquisition were defined using negative and positive controls and kept unchanged for acquisition of all images.

### Visium spatial transcriptomics

#### Mouse brain section preparation for Visium analysis

Tissue sections were prepared according to the standard Visium tissue preparation guide (10x Genomics, CG000408). Slices were mounted on Visium Gene Expression Slides (10x Genomics, 2000233) from the Visium Spatial for FFPE Gene Expression Kit (10x Genomics, PN-1000185, mouse transcriptome). Brains of one mouse at E17.5, P2, P12, and P22 perfused with PFA 4% and postfixed for 48 hours were used. Sections were selected to match to the microdissection area used for the scRNA-seq.

#### Tissue staining, imaging, and Visium library preparation

Tissue slices were processed according to the standard Deparaffinization and H&E Staining and Decrosslinking protocol (10x Genomics, CG000409). Briefly, slices were deparaffinized and stained with hematoxylin, followed by bluing and eosin staining. Slices were covered with mounting medium (85% glycerol) and coverslipped before imaging. Slides were imaged using the Pannoramic SCAN slide scanner (3D Histech) controlled by the Pannoramic Scanner Software. Straight after imaging, coverslips were removed and samples were directly processed according to the standard Visium Spatial Gene Expression protocol (10x Genomics, CG000407) using the Visium Spatial Gene Expression Slide and Reagent Kit (10x Genomics, PN-1000185). Decross-linking was then performed, and probes were immediately hybridized, ligated, released, and collected for library construction following manufacturer’s recommendations (10x Genomics, CG000409). Tissue-covered spots were quantified, and libraries were pooled according to their concentration and spot occupation on slides. Library pool was sequenced using NovaSeq 6000 (Illumina) using the recommended parameters: read 1, 28 cycles; i7 index, 10 cycles; i5 index, 10 cycles; and read 2, 50 cycles.

#### Visium spatial transcriptomics data analysis

Space Ranger version 1.3.0 was used to align reads on the mouse reference genome GRCm38 mm10 and to produce the count matrix. We achieved more than 8000 median genes per spot for each time points. Data analysis was done using Seurat (*60*) for count correction and using the SCTransform() function and SCUtility (*68*) for visualizing the gene expression per spot. We cropped the images of each section with the CropImages() function around the ventricle and selected the V-SVZ corresponding spots. The FeatureOverlay() was finally used to export a png image for all detected genes.

### Electroporations

The following plasmids were used in this study: pCX-GFP (gift of X. Morin, ENS, Paris, France), pPB-CAG-EmGFP (VB161220-1119syh; VectorBuilder Inc., Cyagen Bioscience, Santa Clara, CA, USA), pCMV-Hopx (Open Biosystems, MMM1013-202767606), pCMV-hyPBase (gift of L. Lόpez-Mascaraque, Instituto Cajal, Madrid, Spain), and pcDNA3-CAG-dnALK3 and pcDNA3-CAG-caALK3 [gift of K. Miyazono, University of Tokyo, Japan, corresponding to a dominant-negative form of Bmpr1a that lacks its intracellular domain (i.e., ∆ Bmpr1a) and a constitutively active form (i.e., CA Bmpr1a), respectively]. Plasmids were purified using the EndoFree Plasmid Kit according to the manufacturer’s protocol (QIAGEN, 12362). Plasmids were resuspended to a final concentration of 5 μg/μl. For postnatal fate mapping of pallial RG cells, coelectroporation of pPB-CAG-EmGFP and pCMV-hyPBase plasmids was done at E15.5. For Bmpr1a and Hopx manipulation, electroporation was directed toward the embryonic pallium at late embryogenesis time, i.e., E16.5, as previously described (*69*). EdU (50 mg/kg) was injected 24 hours before euthanasia (i.e. P2).

### Figures

Figures were assembled using Adobe Photoshop. Venn diagrams were done using the “Multiple List Comparator” available online (https://molbiotools.com/listcompare.php).

## Notes

View/request a protocol for this paper from *Bio-protocol*.
