# Supplementary material for: Single-cell analysis of the postnatal dorsal V-SVZ reveals a role for Bmpr1a signaling in silencing pallial germinal activity
Source: Sci Adv. 2023 May 5;9(18):eabq7553. doi: 10.1126/sciadv.abq7553 (PMC10162676; doi:10.1126/sciadv.abq7553)
Supplement: Supplementary file 1 — Figs. S1 to S9 Legends for tables S1 to S7 [file sciadv.abq7553_sm.pdf]

Science Advances
AAAS

Supplementary Materials for

Single-cell analysis of the postnatal dorsal V-SVZ reveals a role for Bmpr1a signaling in silencing pallial germinal activity

Guillaume Marcy *et al.*

Corresponding author: Olivier Raineteau, olivier.raineteau@inserm.fr

Sci. Adv. **9**, eabq7553 (2023)
DOI: 10.1126/sciadv.abq7553

The PDF file includes:

Figs. S1 to S9
Legends for tables S1 to S7

Other Supplementary Material for this manuscript includes the following:

Tables S1 to S7

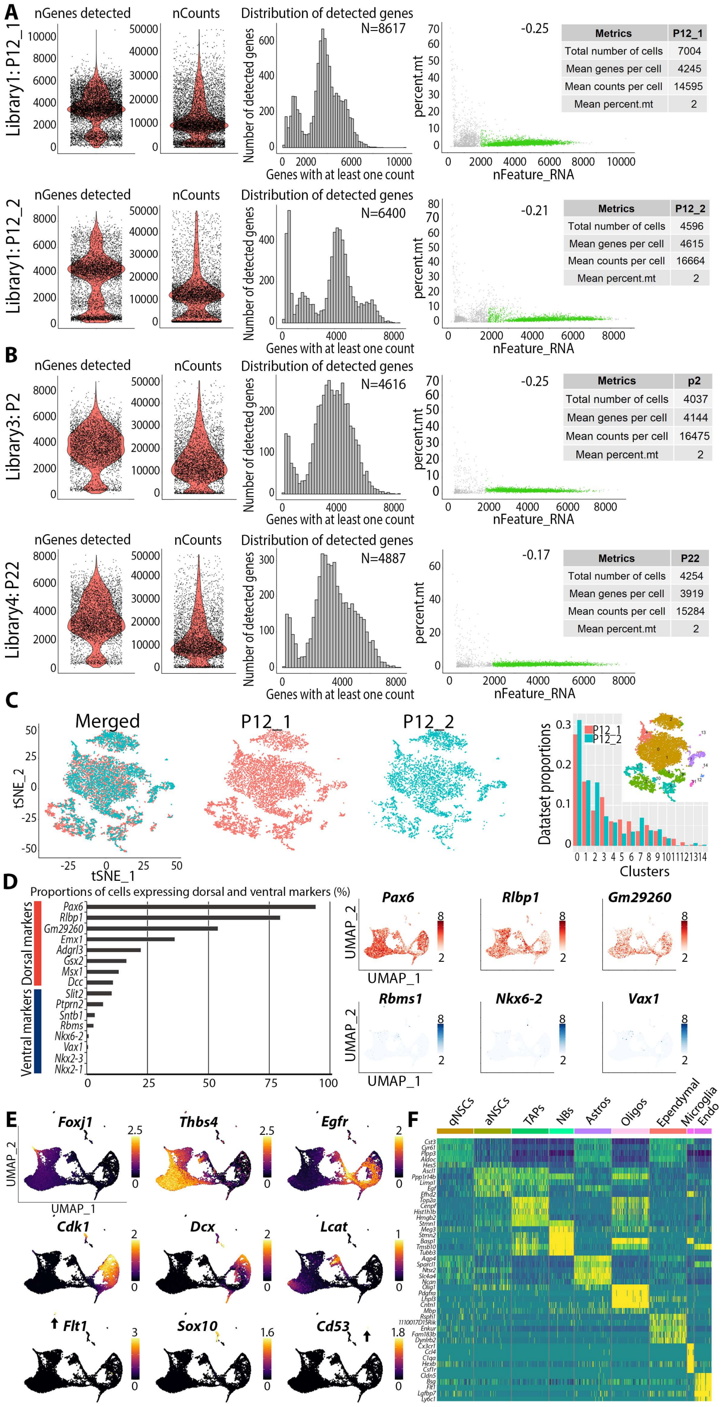

Fig. S1. Data Metrics and Markers, ***related to Fig. 1*****.**

(A) Summary of metrics of the P12 replicates. Cells to be analyzed are selected based on the following criteria: percent.mt<10% and number of genes expressed >2000 & <8000. **(B)** Summary of metrics of the P2 and P22 additional datasets. Cells to be analyzed are selected based on the following criteria: percent.mt<10% and number of genes expressed >2000 & <8000. **(C)** The dimensionality reduction technique tSNE shows the great overlap of the P12 replicates. Their homogenous contribution to the 15 clusters are shown in percentage. **(D)** Percentage of cells expressing *dorsal* and *ventral* V-SVZ markers, and feature plots of most selected genes illustrating the precision of the microdissection approach. **(E)** Markers of the

main cell types observed in the dataset are shown as feature plots. **(F)** Heatmap depicting expression of top five markers distinguishing cell types.

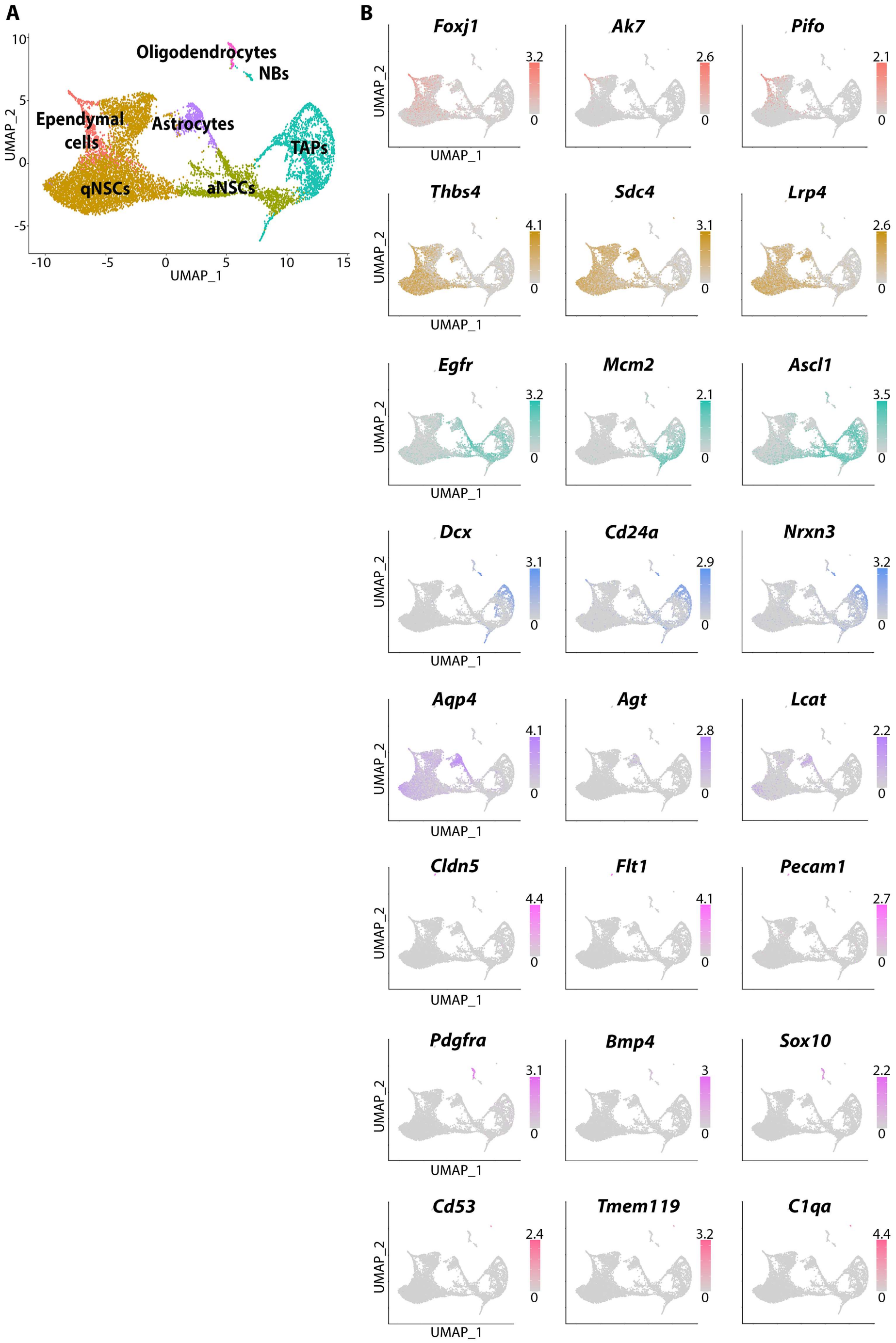

Fig. S2. Markers defining cell types of the dorsal V-SVZ, *related to Fig. 1*.
(A) UMAP with simplified identity annotations. **(B)** Feature plots of markers used to define cell types. Note the overlap of aNSCs markers with TAPs.

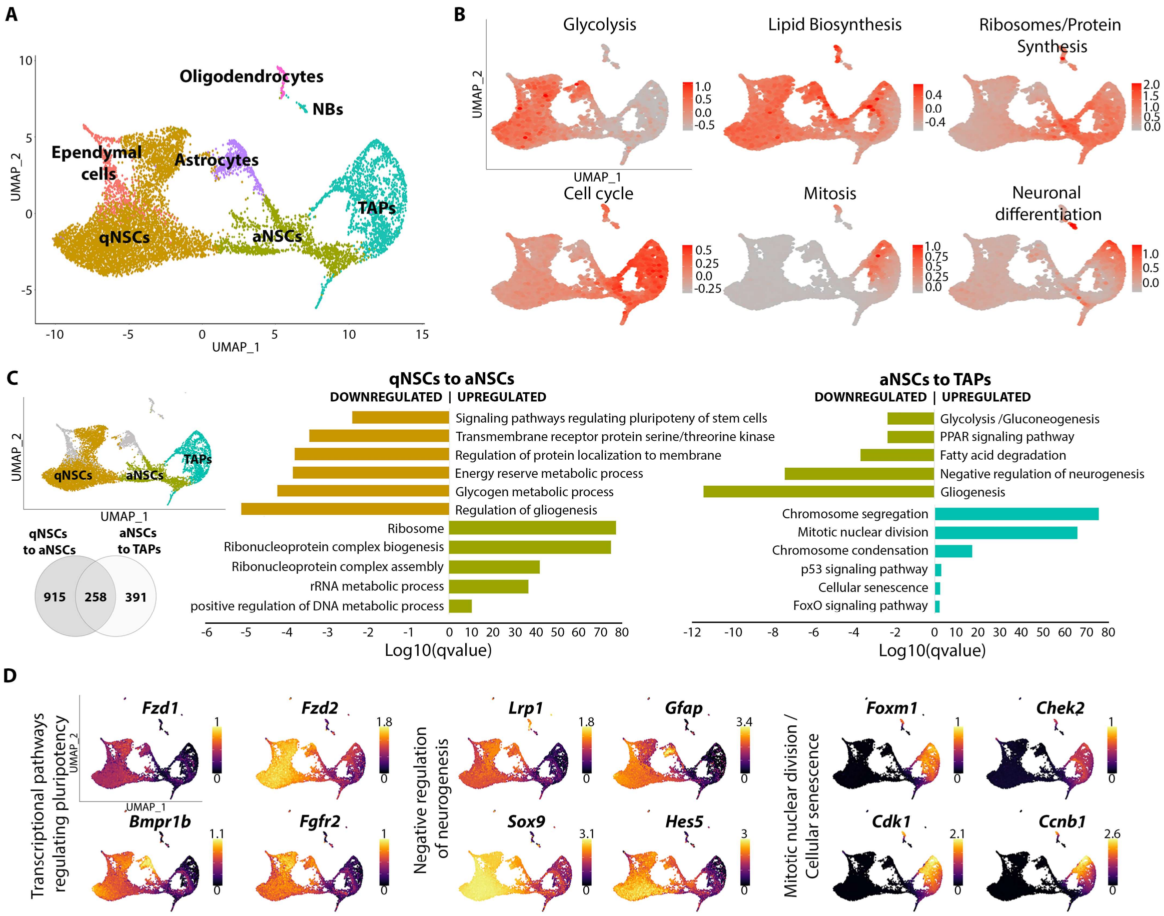

Fig. S3. A core set of "generic genes" defines distinct cellular states, *related to Fig. 1*.
(A) UMAP with simplified identity annotations. **(B)** Percentage of expression of top 20 genes associated with previously described markers reflecting state transitions along the neurogenic lineage (*10*). Dormancy is associated with high glycolytic and lipid metabolisms. Lipogenesis has recently emerged as a key metabolic pathway in hippocampal NSC maintenance (*15*). **(C)** Gene ontology and pathway analyses on "generic genes" allowing transition between qNSCs, aNSCs and TAPs. **(D)** Select genes from regulated gene sets illustrating the dynamics of gene expression during differentiation progression. Note the persistent downregulation of genes involved in gliogenesis as well as glycolytic metabolism to allow differentiation progression.

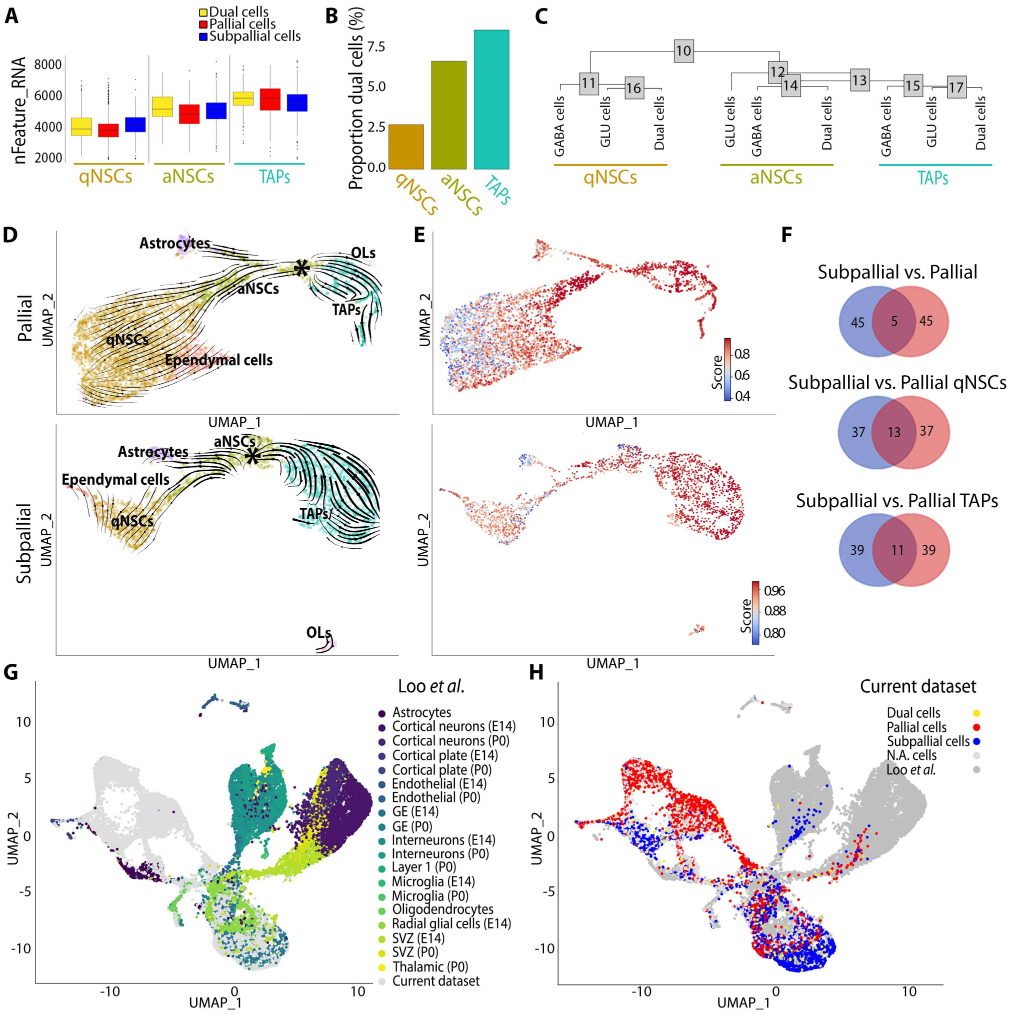

Fig. S4. Pallial and subpallial lineages coexist within the *dorsal* V-SVZ, *related to Fig. 2 & 3*.

(A to C) Dual cells are not doublets as confirmed by a similar average number of detected genes compared to pallial cells and subpallial cells (A). Dual cells proportions within V-SVZ cell types (B). Hierarchical tree shows gradual identity switch of dual cells from a pallial to a subpallial identity (C). **(D to E)** RNA velocity analysis (stochastic model – scVelo) highlights the trajectories cells of the pallial (top) and subpallial (bottom) lineages (D), with high confidence values (E). NBs not included. **(F)** Ven diagram illustrating the minimal overlap of top 50 genes contributing to the velocity calculated in both lineages, qNSCs or TAPs. **(G and H)** UMAP plots of integrated datasets presented in Fig. 3A, with identities of Loo datasets (G), and of pallial and subpallial cells from current P12 dataset (H).

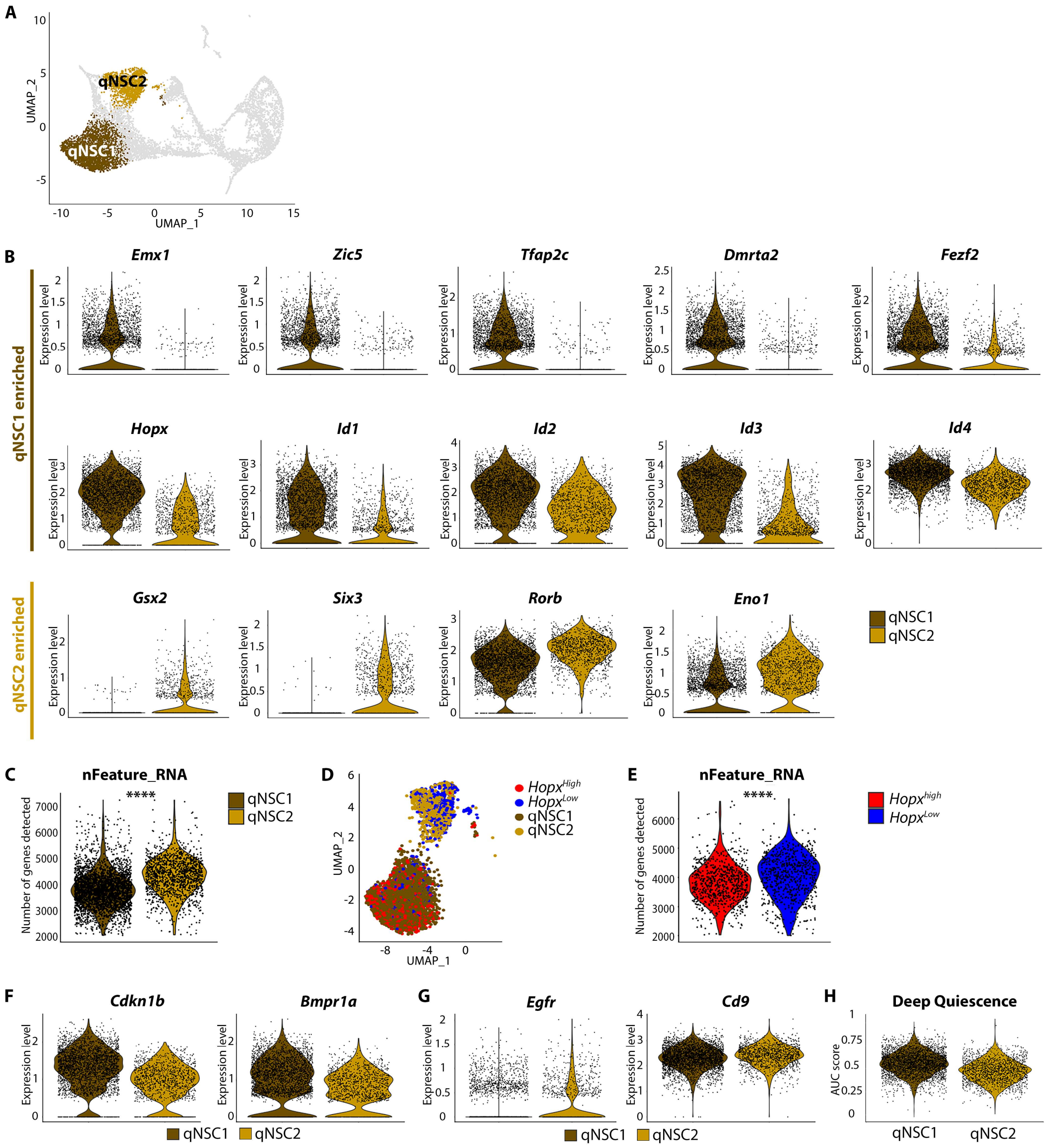

Fig. S5. Pallial and subpallial qNSCs differ in their transcriptional profile and transcript content, *related to Fig. 4.*

(A) UMAP plot highlighting qNSC1 and qNSC2 subclusters. **(B)** Violin plots illustrating expression of transcription factors/transcriptional regulators enriched in qNSC1 and qNSC2. **(C)** Violin plots illustrating the lower transcript content of qNSC1 when compared qNSC2. **(D)** UMAP plot showing enrichment of *Hopx*^{Hght} cells in qNSC1, while *Hopx*^{Low} cells are mainly associated to qNSC2. **(E)** Violin plot showing accordingly higher transcript content in *Hopx*^{Low} cells when compared to *Hopx*^{High} cells. **(F to H)** Violin plots illustrating the enrichment of genes associated to deep quiescence (F) or primed quiescence (G), and calculation of a "deep quiescence" AUC score (H) using markers defined by (*25*).

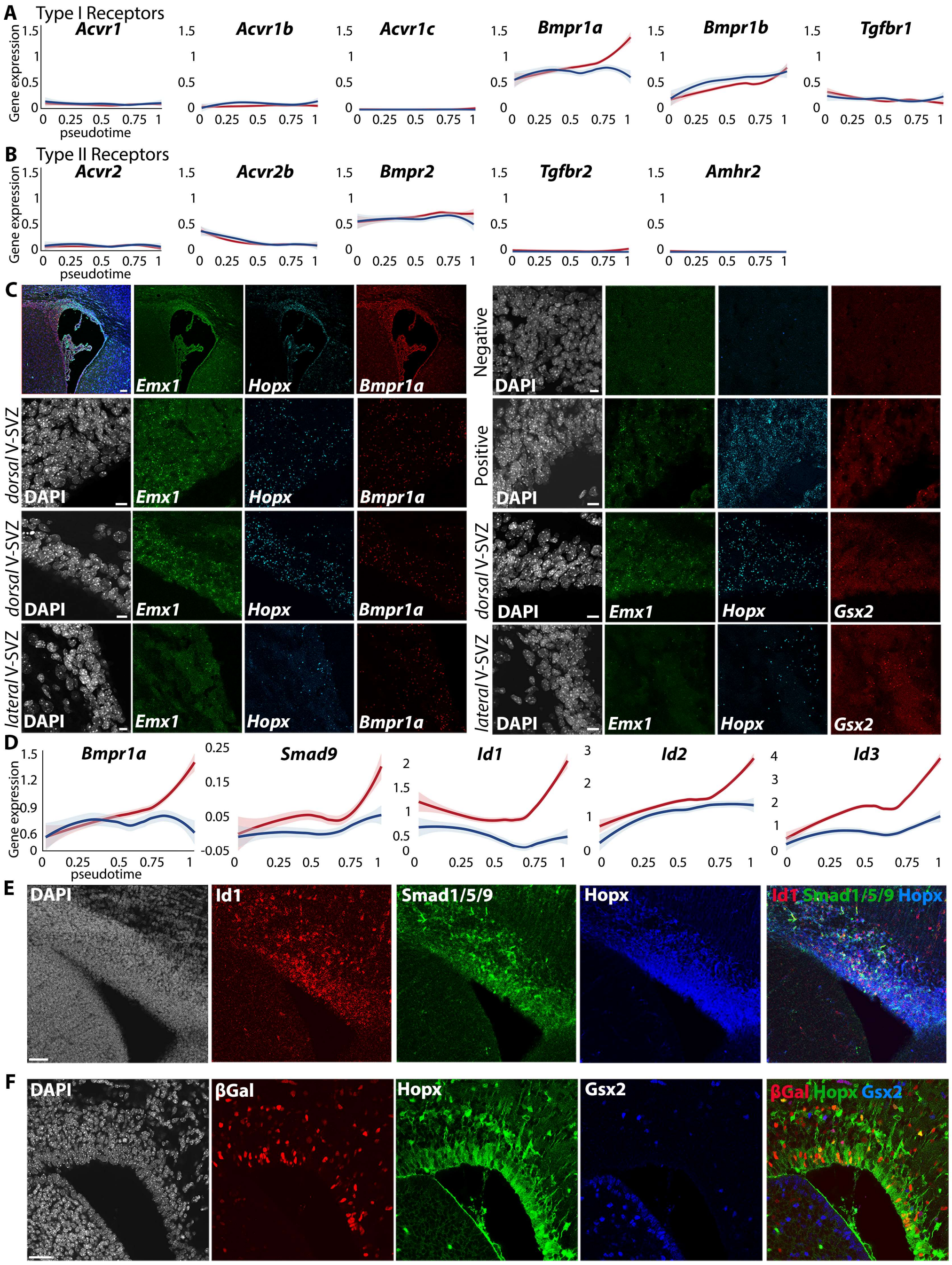

Fig. S6. The *dorsal* V-SVZ shows high TGFb/Wnt signaling activity and contains pallial qNSCs showing high *Bmpr1a* receptor expression and signaling, *related to Fig. 5.*
(A and B) Pseudotime analysis of type I receptors (A) and type II receptors (B) expression in the pallial and subpallial trajectories. **(C)** Expression of *Emx1* (green), *BmpR1a* (red), *Gsx2* (red) and *Hopx* (blue) RNA in the *dorsal* and *lateral* V-SVZ with positive and negative controls of the experiment, shown by RNAScope. **(D)** Pseudotime analysis of *Bmpr1a* and downstream effector (*Smad9*) and target genes (*Id1/2/3*) expression in the pallial and subpallial quiescence trajectories. **(E)** IHC for BMP-signaling effector Smad1/5/9, its downstream target Id1 and the qNSC pallial marker Hopx. **(F)** IHC for the canonical Wnt pathway reporter BAT-gal, the qNSC pallial marker Hopx and the subpallial marker Gsx2. Note the dorsal activity of both

BMP and Wnt canonical signaling within the postnatal V-SVZ. Scale bars: 50µm (overview, C); 10µm (C); 50µm (E & F).

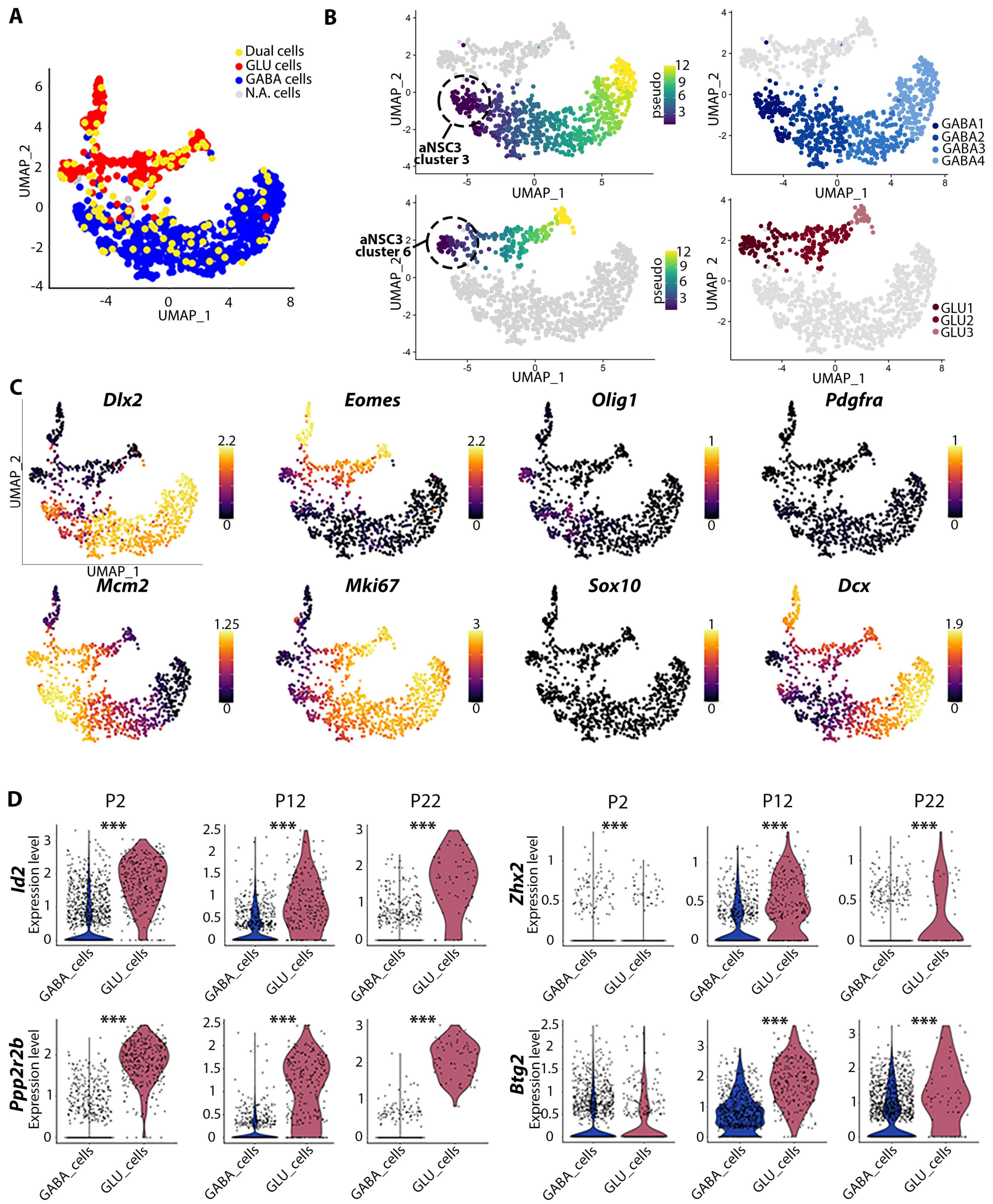

Fig. S7. characterization of GLU and GABA cells within the neuronal trajectory, *related to Fig. 5.*

(A) UMAP plots highlighting distribution of GLU, GABA and Dual cells within the neuronal trajectory. **(B)** Pseudotime analysis within cycling GLU and GABA cells, and related subclusters. **(C)** Feature plots illustrating expression of various cell types or cell status markers. **(D)** Violin plots illustrating selected genes from representative GO terms over-represented in GLU cells at P2, P12 and P22.

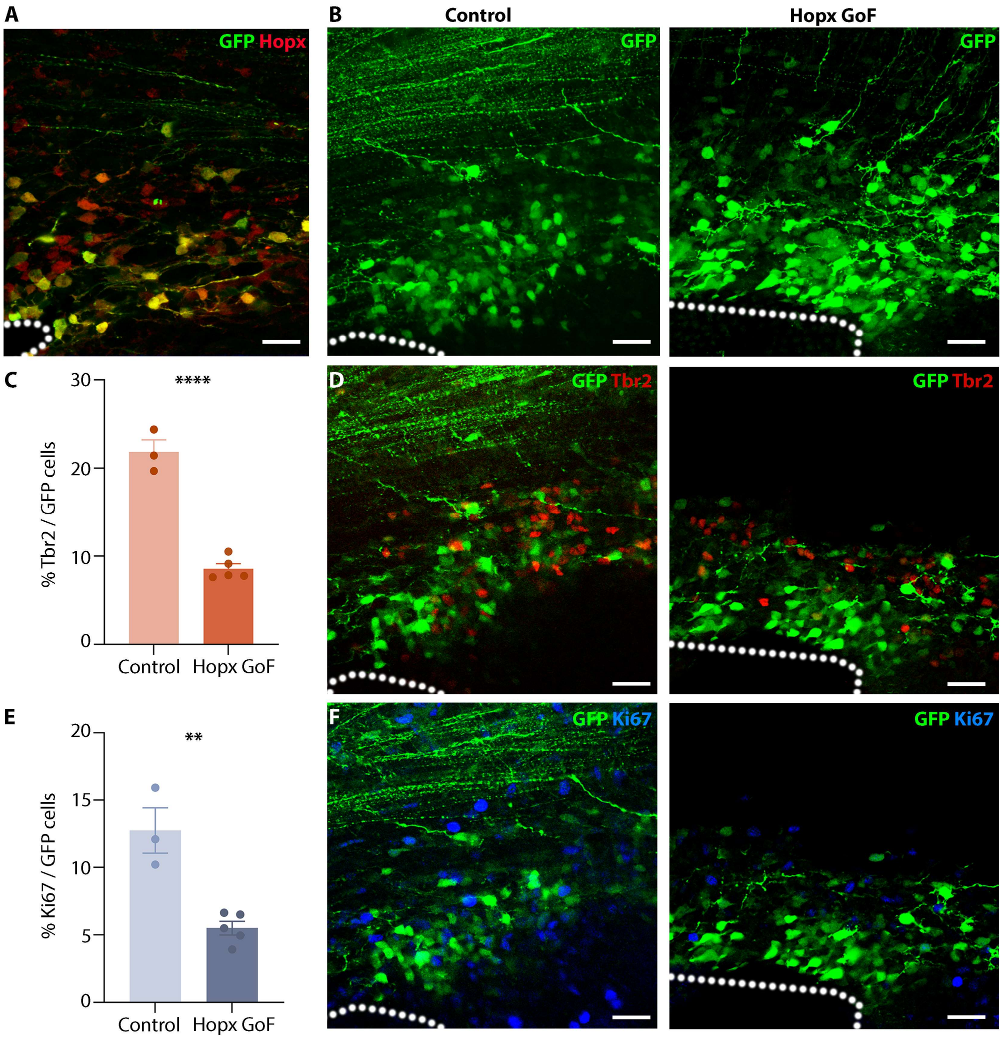

Fig. S8. Hopx gain-of-function reproduces the phenotype observed following CA Bmpr1a overexpression, *related to Fig. 7.*

(A) Representative images of the *dorsal* V-SVZ at P3, electroporated with Hopx plasmid at E16.5. Note the intense Hopx expression in most electroporated (GFP+) cells, while surrounding GFP- cells express lower Hopx levels. The location of the ventricular lumen is indicated by a dotted line. **(B)** Representative overviews of V-SVZ and overlying corpus callosum (CC) in control and Hopx GoF mice. Note the similarity of the Hopx GoF phenotype with the one observed following CA Bmpr1a overexpression, i.e. accumulation of cells showing a radial morphology within the V-SVZ at P3 and delay of axon growth within the CC. **(C to F)** Graphs showing the % of GFP+ electroporated cells expressing Tbr2 (C) or Ki67 (E) and illustrative IHC for the pallial progenitor (Tbr2, red, D) and proliferative (Ki67, blue, F). N = 3 and 5 for control and GoF respectively. Unpaired t-test was performed. Error bars indicate the SEM. Scale bars: 20µm.

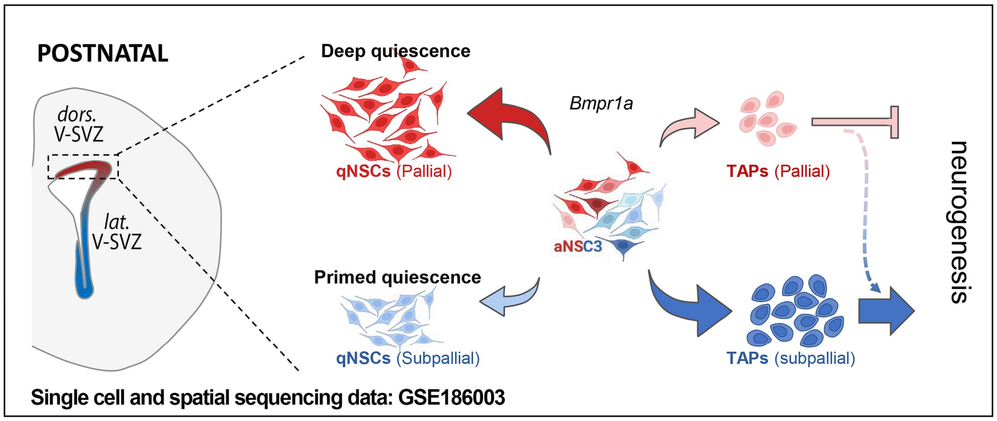

Fig. S9. Graphical abstract summarizing the main findings of this study. Dors V-SVZ, lat V-SVZ: *dorsal* and *lateral* ventricular-subventricular zone, respectively.

TABLES

Table S1: ORA_subclusters_aNSCs. Gene ontology analysis (simplified biological processes) for genes enriched in all aNSCs subclusters. Related to Fig. 2C.
Table S2: aNSC3_subclusters_markers. Genes enriched in aNSC3 subclusters. Related to Fig. 2D&E.
Table S3: ORA_ HopxHigh_vs_Low_qNSCs. Over-representation analysis for GO simplified biological processes, KEGG and Reactome pathways in qNSCs showing high or low levels of *Hopx* expression. Related to Fig. 5A.
Table S4: GSEA_HopxHigh_vs_Low_qNSCs. Ranked gene list and GSEA reports for gene sets enriched in $\textit{Hopx}^{\textit{High}}$ and $\textit{Hopx}^{\textit{Low}}$ qNSCs. Related to Fig. 5B.
Table S5: GSEA_pallial_vs_subpallial_qNSC_trajectories. Curated gene set enrichment analysis performed at 1st and 2nd transition steps of the pallial and subpallial qNSCs trajectories. Related to Fig. 5E.
Table S6: ORA_GLU_vs_GABA_cells. Gene ontology analysis (simplified biological processes) for genes enriched in GLU or GABA cells. Related to Fig. 6E&F.
Table S7: Markers_of_signatures. Gene contributing to signatures used in specified analyses.
